# The Development of Robust Antibodies to Sarcospan, a Dystrophin- and Integrin-Associated Protein, for Basic and Translational Research

**DOI:** 10.3390/ijms25116121

**Published:** 2024-06-01

**Authors:** Ekaterina I. Mokhonova, Ravinder Malik, Hafsa Mamsa, Jackson Walker, Elizabeth M. Gibbs, Rachelle H. Crosbie

**Affiliations:** 1 Department of Integrative Biology and Physiology, University of California Los Angeles, Los Angeles, CA 90095, USA; 2 Eli and Edythe Broad Center of Regenerative Medicine and Stem Cell Research, University of California Los Angeles, Los Angeles, CA 90095, USA; 3 Molecular Biology Institute, University of California Los Angeles, Los Angeles, CA 90095, USA; 4 Department of Neurology, David Geffen School of Medicine, University of California Los Angeles, Los Angeles, CA 90095, USA

**Keywords:** antibody, dystrophin, monoclonal, muscular dystrophy, polyclonal, sarcoglycan, sarcospan, skeletal muscle

## Abstract

Sarcospan (SSPN) is a 25-kDa transmembrane protein that is broadly expressed at the cell surface of many tissues, including, but not limited to, the myofibers from skeletal and smooth muscles, cardiomyocytes, adipocytes, kidney epithelial cells, and neurons. SSPN is a core component of the dystrophin–glycoprotein complex (DGC) that links the intracellular actin cytoskeleton with the extracellular matrix. It is also associated with integrin α7β1, the predominant integrin expressed in skeletal muscle. As a tetraspanin-like protein with four transmembrane spanning domains, SSPN functions as a scaffold to facilitate protein–protein interactions at the cell membrane. Duchenne muscular dystrophy, Becker muscular dystrophy, and X-linked dilated cardiomyopathy are caused by the loss of dystrophin at the muscle cell surface and a concomitant loss of the entire DGC, including SSPN. SSPN overexpression ameliorates Duchenne muscular dystrophy in the *mdx* murine model, which supports SSPN being a viable therapeutic target. Other rescue studies support SSPN as a biomarker for the proper assembly and membrane expression of the DGC. Highly specific and robust antibodies to SSPN are needed for basic research on the molecular mechanisms of SSPN rescue, pre-clinical studies, and biomarker evaluations in human samples. The development of SSPN antibodies is challenged by the presence of its four transmembrane domains and limited antigenic epitopes. To address the significant barrier presented by limited commercially available antibodies, we aimed to generate a panel of robust SSPN-specific antibodies that can serve as a resource for the research community. We created antibodies to three SSPN protein epitopes, including the intracellular N- and C-termini as well as the large extracellular loop (LEL) between transmembrane domains 3 and 4. We developed a panel of rabbit antibodies (poly- and monoclonal) against an N-terminal peptide fragment of SSPN. We used several assays to show that the rabbit antibodies recognize mouse SSPN with a high functional affinity and specificity. We developed mouse monoclonal antibodies against the C-terminal peptide and the large extracellular loop of human SSPN. These antibodies are superior to commercially available antibodies and outperform them in various applications, including immunoblotting, indirect immunofluorescence analysis, immunoprecipitation, and an ELISA. These newly developed antibodies will significantly improve the quality and ease of SSPN detection for basic and translational research.

## 1. Introduction

The SSPN protein forms homo-oligomers at the cell surface to create a scaffold for many cell surface receptors that interact with the extracellular matrix. The expression of SSPN enhances cell adhesion, cell signaling, and mechanotransduction [1,2]. SSPN interacts tightly with many multimeric transmembrane protein complexes, including the dystrophin–glycoprotein complex (DGC), the utrophin–glycoprotein complex (UGC), and the α7β1–integrin complex [3,4]. Within the DGC and UGC, SSPN mediates protein–protein interactions between the dystroglycan (α- and β-subunits) and the sarcoglycan subcomplex, an interaction that is critical for membrane targeting and the stable interaction with laminin [4]. In the skeletal muscles, SSPN is enriched at the myotendinous junction and the neuromuscular junction, where it interacts with ECM receptors and mechanotransducers [4]. Many forms of muscular dystrophy result from a loss of muscle cell attachment to the extracellular matrix [5]. For instance, perturbations of the sarcoglycan–SSPN subcomplex are associated with autosomal recessive limb–girdle muscular dystrophy (LGMD) [6]. It is well established that stable interactions among the integral membrane proteins are critical for the function of adhesion complexes and the prevention of muscular dystrophy [7]. Despite their importance, the factors determining the structural integrity of the DGC, UGC, and α7β1–integrin complex are incompletely understood.

We have previously shown that SSPN is absent in muscle biopsies from Duchenne muscular dystrophy (DMD) patients and is lost in LGMD patients with complete or partial absence of the sarcoglycan complex [8]. Consistent with these clinical observations, SSPN is absent from the sarcolemma of four different sarcoglycan-deficient murine models of LGMD (types R3, R4, R5, and R6) caused by mutations in genes encoding any one of the four canonical sarcoglycan proteins (α-, β-, γ-, and δ-sarcoglycan) [7,9,10,11]. The transgenic overexpression of SSPN in *mdx* mice, a murine model of DMD, increases the abundance of the compensatory ECM receptors—the UGC and α7β1–integrin complex—at the myofiber membrane [12]; increases the C-type 2 glycosylation of α-DG [13]; and enhances laminin binding [12,14,15].

Our studies exploring the therapeutic benefit of the SSPN-based sarcolemma stabilization of DMD skeletal and cardiac muscle have revealed that SSPN overexpression effectively improves numerous aspects of DMD pathology [12,14,16,17]. Even low levels of SSPN overexpression can enhance *mdx* cardiac membrane stability, upregulate utrophin expression, and improve cardiac function [18], in addition to addressing skeletal muscle pathology and the contraction-induced injury to myofibers [13,16,18,19]. Based on these studies, we have conducted high-throughput screening to identify the small molecules that enhance SSPN expression in mature muscle cells [20,21,22]. We identified and validated the lead compounds that increased SSPN gene and protein expression in dystrophin-deficient mouse and human muscle cells [21,22]. The lead compounds increase the cell membrane localization of compensatory laminin-binding adhesion complexes and improve membrane stability in DMD myotubes. Using siRNAs to knock down SSPN before the compound treatment, we demonstrated that the compound’s membrane stabilizing benefit depends on SSPN expression [21]. Our laboratory continues its efforts to develop small molecule enhancers for treating DMD.

The availability of specific and robust anti-SSPN antibodies is vital for evaluating the efficiency of SSPN-modulating therapies. In the current report, we describe the generation of anti-SSPN monoclonal and polyclonal antibodies to three regions of the polypeptide: the N-terminus, the large extracellular loop (LEL; between transmembrane domains 3 and 4), and the C-terminus. We tested antibody specificity to SSPN in many commonly used assays, including immunoblotting (IB), immunofluorescent analysis (IFA), immunoprecipitation (IP), and an enzyme-linked immunosorbent assay (ELISA). We provide a summary of the performance of all antibodies, revealing that our specific, high-titer antibodies address the obstacles with commercially available antibodies, thus providing a resource that is unavailable elsewhere.

## 2. Results

**Figure 1:**
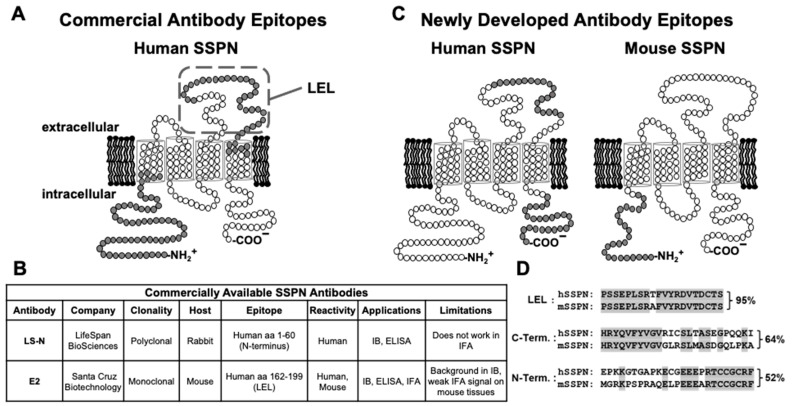
SSPN epitopes are used for antibody development. (**A**) Schematic diagram representing the predicted membrane topology of human SSPN protein and the corresponding epitopes of commercially available antibodies (in gray). (**B**) Summary of commercially available antibodies, their epitopes, applications, and limitations. (**C**) Schematic diagrams representing the predicted membrane topology of mouse (mSSPN) and human (hSSPN) SSPN proteins and the corresponding epitopes of newly developed antibodies (in gray). hSSPN has 243 amino acids, while mSSPN has 216, with the difference mostly being due to the longer N-terminus of hSSPN. (**D**) Comparison of the amino acid residues of hSSPN and mSSPN in the epitope regions used to immunize rabbits and mice. The amino acid sequence is shown in a single-letter code. Non-matching amino acids between hSSPN and mSSPN are labeled by gray in the amino acid sequence of mSSPN.

SSPN plays a scaffolding role in facilitating protein–protein interactions within adhesion complexes and can serve as a biomarker for the proper assembly and membrane expression of the DGC [3,4,6,7]. Our lab is pursuing the development of SSPN-based therapy to treat DMD and LGMD muscular dystrophies caused by deficits in adhesion proteins. Highly specific and robust anti-SSPN antibodies are vital for evaluating the efficiency of SSPN-modulating therapies, pre-clinical studies, and basic research focused on the molecular mechanisms of SSPN-mediated rescue. Most commercial antibodies to SSPN are generated by antigens to human SSPN (hSSPN) and have a limited range of applications (Figure 1A–C; LS-N antibody as an example) which is a major limitation to pursuing basic and translational research in the muscular dystrophies. In fact, only a few of the currently available antibodies to hSSPN cross-react with murine SSPN (mSSPN) (Figure 1C; E2 antibody as an example) Furthermore, the mSSPN epitope is located within the intracellular N-terminus (Figure 1A,B), which requires fixation and membrane permeabilization for detection in cell culture experiments. While commercial antibodies to SSPN can be convenient, the range of epitopes within SSPN, the extent of species cross-reactivity, and the flexibility for multiple applications are underdeveloped.

In this study, we sought to create a panel of monoclonal and polyclonal antibodies to address these barriers and to complement the existing commercial antibodies. In order to define the specific polypeptides to target, we conducted B-cell prediction analysis and found that only about 30% of the amino acid sequence of both mSSPN (Supplementary Figure S1A) and hSSPN (Supplementary Figure S1B) can be used for antibody development. Over 70% of SSPN polypeptide is buried in the membrane bilayer by virtue of its four transmembrane helices, which leaves the N- and C-termini as well as the extracellular large extracellular loop (LEL) located between transmembrane domains 3 and 4 as the available antigenic sites. To maximize potential species cross-reactivity, we targeted a region in the LEL that is 95% identical between the mSSPN and hSSPN proteins for immunization (Figure 1C,D). The N- and C-termini of the mSSPN and hSSPN are highly species-dependent (Figure 1C), suggesting that any polypeptide selected within these regions would likely be species-specific. Since a polyclonal antibody to the hSSPN N-terminus is commercially available, we selected a human specific C-terminal peptide with 64% identity to mSSPN as a target antigen (Figure 1C,D). We then identified a murine-specific fragment within the C-terminus that exhibits a 52% amino acid identity to hSSPN (Figure 1C,D).

As it is well recognized that creating antibodies is resource- and time-intensive, in addition to being unpredictable, we crafted a strategy that would complement existing commercial antibodies in terms of coverage across distinct regions of the protein, multiple species cross-reactivity, and antibody diversity with the goal of maximizing SSPN antibodies as tools for research and clinical applications. The antibodies produced in mice are usually suitable for common applications such as IB, an ELISA, flow cytometry, and IP. However, they are less likely to produce satisfying results for staining applications such as immunohistochemistry (IHC) and immunocytochemistry (ICC), especially when used on mouse tissue samples. As part of our antibody diversity strategy, we decided to create monoclonal antibodies in mice and in rabbits. Relative to the murine hosts, the rabbit antibody hosts exhibit a high success rate with a larger range of epitopes, including challenging antigens such as small molecules, peptides, and post-translational modification (PTM) sites that are often non-immunogenic in mice. While prediction tools have increased the likelihood of generating antibodies usable for research applications, the animal-to-animal variability in immune response can be unpredictable. For this reason, we elected to immunize several individual rabbits for the polyclonal antibody production.

### 2.1. Development of Rabbit Polyclonal Antibody to N-Terminus of Mouse SSPN

**Figure 2:**
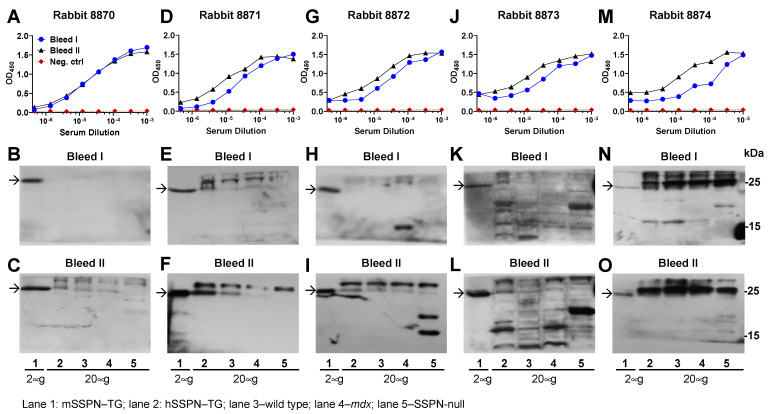
Testing rabbit immune response to mSSPN with the ELISA and immunoblotting. **Panels** (**A**,**D**,**G**,**J**,**M**): mSSPN-specific rabbit sera titration curves with the ELISA. The blood was collected seven days after the second (bleed I) and third (bleed II) booster doses. Serial dilutions of each rabbit serum were added to the ELISA plates coated with SUMO-tagged mSSPN N-terminus (MGRKPSPRAQELPEEEARTCCGCRF, U02487). HRP-conjugated goat anti-rabbit IgG was used to detect the mSSPN-specific rabbit IgG. TMB was used as a substrate for the HRP. The enzymatic reaction was stopped by 2 M of sulfuric acid, and the plates were read at 450 nm. **Panels** (**B**,**C**,**E**,**F**,**H**,**I**,**K**,**L**,**N**,**O**): Immunoblotting of bleeds I and II from each rabbit. Rabbit 8870—panels (**B**,**C**); rabbit 8871—panels (**E**,**F**); rabbit 8872—panels (**H**,**I**); rabbit 8873—panels (**K**,**L**); and rabbit 8874—panels (**N**,**O**). Immunoblotting of bleeds I and II with total muscle lysates from mice overexpressing mouse SSPN (mSSPN-TG, arrow) (lane 1), human SSPN (hSSPN-TG) (lane 2), wild type (lane 3), *mdx* (lane 4), and SSPN-null (lane 5). m-SSPN-TG, wild type, and *mdx* lysates were used for the evaluation of the specific immune response to mouse SSPN. h-SSPN-TG lysate was used for detection of possible cross-reactivity between the SSPN of mouse and human origins. The SSPN-null lysate was used to exclude rabbits generating a non-specific response. mSSPN was detected at 25 kDa (arrow) by immunoblotting. The skeletal muscle was solubilized using an RIPA buffer, and the protein samples (20 μg of total protein per line for all lines, except mSSPN-TG (lane 1) where 2 μg of total protein per line was loaded) were separated by SDS-PAGE and transferred to nitrocellulose membranes. Immunoblots were probed with crude non-purified rabbit sera diluted 1:150 (bleed I) or 1:600 (bleed II) in the blocking buffer. Arrows indicate the mSSPN. The ~27 kDa bands presenting in all muscle lysates (panels (**C**,**E**,**F**,**I**,**L**,**N**,**O**)) were not SSPN-related and may represent secondary antibody cross-reactivity with endogenous antibody light chains in the skeletal muscle protein lysates.

We first developed rabbit polyclonal antibodies using a recombinant GST fusion protein fused to an N-terminal fragment of mouse SSPN (mSSPN, aa 1–25, MGRKPSPRAQELPEEEARTCCGCRF, U02487) (Figure 1C,D). This fragment was chosen based on the results of the mSSPN antigenic epitope prediction (Supplementary Figure S1A) and our prior observations of a robust immune response to this region [4,8,12,13]. Five New Zealand white rabbits were immunized with a GST N-terminal fragment of mSSPN fused with GST (Rabbits #8870, #8871, #8872, #8873). After two booster doses (bleed I), all rabbits developed strong immune responses to the mSSPN protein, and sera from all rabbits recognized the mSSPN in the ELISA at dilutions ranging from 3 × 10^−5^ to 3 × 10^−6^ (Figure 2A,D,G,J,M). A side-by-side comparison of the rabbit sera after subsequent boosters (bleeds I and II) revealed that rabbits #8871, #8872, #8873, and #8874 had an enhanced response to recombinant SSPN after the third booster dose (bleed II) (Figure 2D,G,J,M). Rabbit #8870 developed a robust immune response after the second boost, and this response was maintained at the same level after the third boost (Figure 2A). To evaluate the specific response to the native SSPN protein, we employed the immunoblotting method where non-purified rabbit sera were blotted with total protein lysates generated from the muscles of wild type, *mdx*, mouse SSPN-TG, human SSPN-TG, and SSPN-null mice (Figure 2B,C,E,F,H,I,K,L,N,O). These lines exhibit various levels of mSSPN and hSSPN protein expression (Table 1) from no expression in the SSPN-nulls [23] and reduced expression in the *mdx* (5~10-fold relative to wild-type levels) [12] to high-level expression in both the mouse SSPN-TG and human SSPN-TG lines [17].

We detected endogenous mSSPN in the mSSPN-TG lysate, where SSPN is the most highly expressed, in the sera from all rabbits after the second booster (bleed I) (Figure 2). Subsequent boosters (bleed II) increased the immune response to mSSPN in all five rabbits (Figure 2). A specific signal with endogenous mSSPN in the wild type, hSSPN-TG, and *mdx* samples was observed after the third boost (bleed II) in rabbits #8870, #8871, and #8872 (Figure 2A,D,G). The band observed in the hSSPN-TG samples (lane 2 on the two lower immunoblotting panels) represents endogenous mSSPN in the hSSPN-TG muscles. The sera from rabbits # 8873 and 8874 generated a high non-specific background and a very low signal with the protein of interest (lane 1), including the prominent IgG light chain bands running at a similar molecular weight with SSPN (25 kD) in all tested samples, including the SSPN-null mice (lane 5) with immune sera from rabbit #8874 (Figure 2N,O).

Bleeds II from all rabbits were also analyzed by an indirect IFA on the skeletal muscle cryosections. A positive signal in the mSSPN-TG and WT muscles was observed with the sera from rabbits #8870 to #8873 (Supplementary Figure S2). The serum from rabbit #8874 did not recognize the mouse SSPN in the indirect IFA and produced the highest background in immunoblotting, leading to the exclusion of this rabbit from further antibody development. Rabbit #8873 was also excluded from extended immunization because of the non-specific bands observed in the immunoblots of the murine skeletal muscle lysates with bleeds from this rabbit (Figure 2K,L). However, serum #8873 recognized exogenous and endogenous SSPN in mSSPN-TG and wild type muscles in the IFA and exhibited no detectable staining in the SSPN-nulls (Supplementary Figure S2). Based on these results from the ELISA, IFA, and immunoblotting, we chose to purify the polyclonal antibodies from the terminal bleed from this rabbit specifically for the IFA applications.

The serum from rabbit #8871 up to a dilution of 3 × 10^−6^ recognized the mSSPN protein in the ELISA after the third boost (Figure 2D) and generated robust, specific signals to endogenous mSSPN with minimal background in the immunoblotting (Figure 2E,F). In the IFA, the serum from this rabbit recognized endogenous mSSPN not only in the WT and SSPN-TG muscle sections but also in the *mdx* sections, where the level of SSPN protein is significantly reduced (Supplementary Figure S2). The serum from rabbits #8870 (Figure 2B,C) and #8872 (Figure 2H,I) recognized the endogenous mSSPN protein in the wild-type, SSPN-TG, and *mdx* samples in the immunoblotting and the wild-type and SSPN-TG samples in the IFA (Supplementary Figure S2). Based on the combined results of the ELISA, IFA and immunoblotting, rabbit #8871 was chosen for the monoclonal antibody production; rabbits #8870 and #8872 were selected for extended immunization and the generation of polyclonal antibodies to mSSPN. These rabbits received three additional booster doses on days 84, 105, and 126 (Supplementary Table S2). A week after each booster, bleeds III–V were collected and analyzed in an ELISA. Both rabbits maintained a stable immune response against the antigens during the extended immunization, with robust reactivity detected even upon serum dilutions in the 10^−6^ range (Supplementary Figure S3).

**Figure 3:**
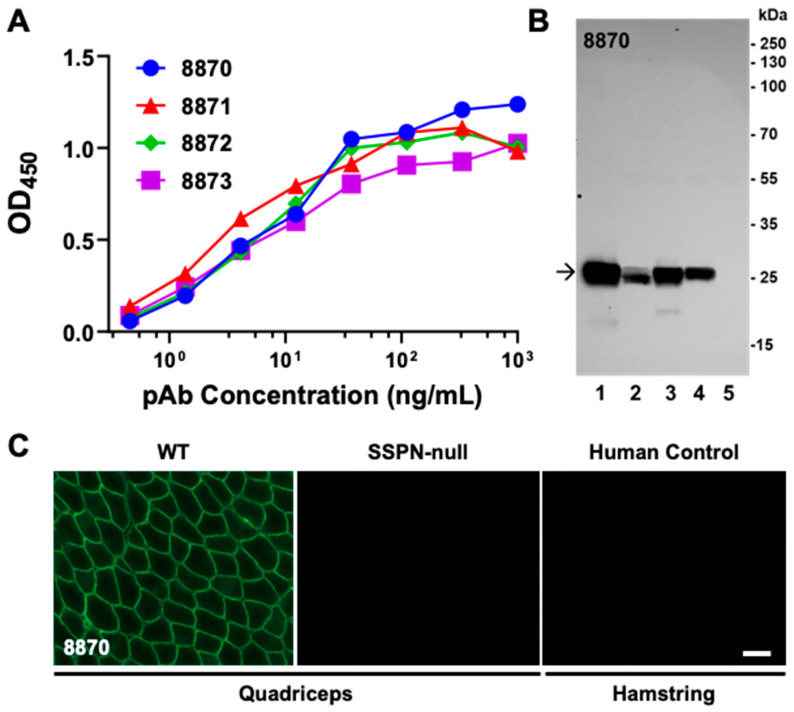
Validation of rabbit polyclonal antibodies to mouse SSPN N-terminus. (**A**) Validation of purified rabbit polyclonal antibodies with an ELISA. Terminal bleeds from rabbits #8870, #8871, #8872, and #8873 were purified by affinity chromatography. The titer of purified antibodies was estimated by the ELISA with SUMO-tagged mSSPN (MGRKPSPRAQELPEEEARTCCGCRF, U02487). HRP-conjugated goat anti-rabbit IgG was used to detect the mSSPN-specific rabbit IgG. TMB was used as a substrate for the HRP. The enzymatic reaction was stopped by 2 M of sulfuric acid, and the plates were read at 450 nm. (**B**) Immunoblotting of the total muscle lysates from the mSSPN-TG (lane 1), hSSPN-TG (lane 2), WT (lane 3), *mdx* (lane 4), and SSPN-null (lane 5) mice with purified rabbit polyclonal antibodies #8870. There was 20 μg of total protein per line for all lines, except mSSPN-TG where 2 μg of total protein per line was loaded. The arrow indicates the mSSPN. Film exposure time, 15 s. (**C**) Immunofluorescence images of the transverse cross-sections of the quadriceps muscles of the WT and SSPN-null mice and the human control (hamstring) stained with purified rabbit polyclonal antibodies #8870. The acquisition time for all muscle cross-sections was 0.8 s. Scale bar, 50 μm.

Polyclonal antibodies were purified from the terminal bleeds of all rabbits, except #8874, and evaluated with an ELISA, IFA, and immunoblotting (Figure 3). The antibodies demonstrated high titers in the ELISA against mSSPN with OD_450_ values above 0.5 R.U. at antibody concentrations of 1 ng/mL (Figure 3A) and recognized the endogenous mouse SSPN with a high affinity and specificity in the immunoblotting and IFA. Representative IFA and immunoblotting images with polyclonal antibodies from rabbit #8870 sera are presented in Figure 3B,C. The rabbit antibodies generate strong bands in immunoblotting with mSSPN across all muscle tissues where this protein is present (wild type, mSSPN-TG, hSSPN-TG, and *mdx*) (Figure 3B), recognize mSSPN at the sarcolemma of muscle fibers (Figure 3C), and do not-cross react with hSSPN in either the IFA or immunoblotting (Figure 3B,C).

### 2.2. Development of Rabbit Monoclonal Antibody to N-Terminus of Mouse SSPN

For the generation of rabbit monoclonal antibodies, single B-cell sorting technology [24] was applied to rabbit #8871. Non-purified immune serum from this rabbit recognized mSSPN with a high affinity and specificity, generating strong specific signals in the ELISA, immunoblotting, and IFA (Figure 2D and Supplementary Figure S2). Multi-parameter flow cytometry single-cell sorting [25,26] was used to isolate antigen-specific IgG^+^/IgM^−^ B cells at a single-cell density. Twenty 96-well plates of B-cells were isolated, providing a potential 1920 clones. The supernatants of all 1920 clones were screened in the ELISA for reactivity to the mSSPN recombinant SUMO-tagged antigen by ABclonal to select the mSSPN-specific clones not cross-reacting with the fusion protein (GST) used for the immunization. The cell supernatants from 96 clones with the highest titers in the ELISA (Supplementary Table S3A) were selected for further evaluation by immunoblotting with the total muscle lysates prepared from the wild type, mSSPN-TG, hSSPN-TG, *mdx*, and SSPN-null mice (Table 1). The antibodies from thirty-nine individual clones were hybridized to endogenous mSSPN in the immunoblotting assays and further evaluated in the IFA (Supplementary Table S3B). Of these, fifteen clones with robust signals in both the immunoblotting and IFA were selected for the next steps of monoclonal antibody development (RNA extraction, RT-PCR, and construction of the LEMs). The LEMs were transiently transfected into HEK 293-F cells, and ABclonal performed a secondary ELISA screening of the supernatants from the transiently transfected cells. Thirteen of the fifteen selected clones retained their anti-mSSPN activity in the ELISA after constructing linear expression cassettes (Supplementary Table S3C). These clones were re-screened with immunoblotting and IFA, and the seven best-performing clones (Table 2) were selected for expression and large-scale purification of monoclonal antibodies. All seven clones retained their activity after large-scale antibody production, producing robust specific signals in the ELISA, IFA, and immunoblotting.

**Figure 4:**
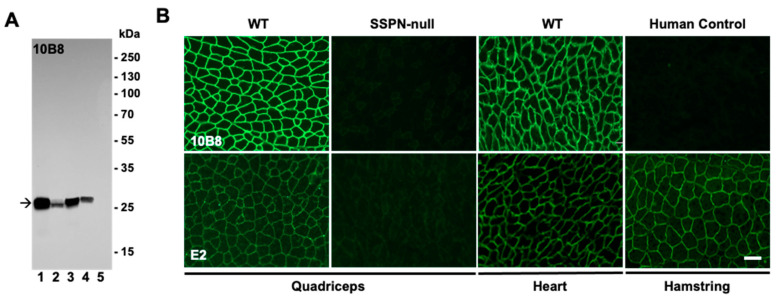
Validation of rabbit monoclonal antibodies to mouse SSPN N-terminus. (**A**) Immunoblotting of total muscle lysates from mSSPN-TG (lane 1), hSSPN-TG (lane 2), WT (lane 3), *mdx* (lane 4), and SSPN-null (lane 5) with newly developed rabbit monoclonal antibody 10B8. There was 20 μg of total protein per line for all lines, except mSSPN-TG where 2 μg of total protein per line was loaded. The arrow indicates the mSSPN. Film exposure time, 15 s. (**B**) Immunofluorescence images of transverse cross-sections of the quadriceps of WT and SSPN-null mice, WT hearts, and the human control hamstring stained with newly developed rabbit monoclonal antibody 10B8 and commercially available mouse monoclonal antibody E2 for comparison. The acquisition time for E2 was 1 s, and for 10B8, it was 0.8 s. Scale bar, 50 μm.

Representative images of the validation of one of the final clones, 10B8, by the IFA and immunoblotting are shown (Figure 4A,B) side-by-side with the commercially available mouse monoclonal antibody E2 (Santa Cruz, sc393187). The immunoblotting revealed tge strong reactivity of 10B8 toward endogenous mSSPN in muscle lysates from wild-type, SSPN-TG, and *mdx* mice without non-specific bands in the SSPN-null lysates (Figure 4A). Our newly developed rabbit antibody, both polyclonal (Figure 3B) and monoclonal (Figure 4A), outperforms E2 in terms of its reactivity and specificity to endogenous mSSPN. Our antibodies exhibited a clean signal without the non-specific bands observed in the immunoblotting with the E2 antibody. A side-by-side comparison of the IFA signals revealed that our rabbit monoclonal antibodies generate stronger signals in the mouse quadriceps and heart compared to E2 under the same experimental conditions (Figure 4B). The rabbit polyclonal and monoclonal antibodies exhibited strong specific signals with the mSSPN protein but not with hSSPN in the immunoblotting and IFA (Figure 3 and Figure 4), supporting the initial hypothesis that our rabbit antibodies are specific to mSSPN, with no cross-reactivity against hSSPN.

### 2.3. Development of Mouse Monoclonal Antibodies to the LEL Fragment and C-Terminus of Human SSPN

**Figure 5:**
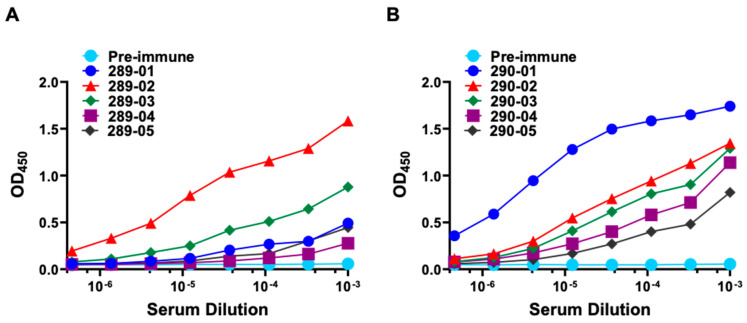
Reactivity of mouse sera to C-terminus and LEL fragment of human SSPN in an indirect ELISA. hSSPN-specific mouse IgG titration curves. Blood was collected seven days after the second (bleed I) booster dose. Serial dilutions of each mouse serum were added to the ELISA plates coated with a biotinylated peptide of hSSPN C-terminus (HRYQVFYVGVRICSLTASEGPQQKI, AF016028) (**A**) or with a biotinylated peptide of hSSPN LEL fragment (PSSEPLSRTFVYRDVTDCTS) (**B**). HRP-conjugated goat anti-mouse IgG was used to detect hSSPN-specific mouse IgG. TMB was used as a substrate for the HRP. The enzymatic reaction was stopped by 2 M of sulfuric acid, and the plates were read at 450 nm.

We developed mouse monoclonal antibodies against two fragments of hSSPN (GenBank accession number AF016028) from the C-terminus region (aa 219–243, RYQVFYVGVRICSLTASEGPQQKI) and a portion of the LEL (aa 167–186, PSSEPLSRTFVYRDVTDCTSC) (Figure 1A,C,D). Five mice per antigen were immunized (Supplemental Table S4). A cohort of mice immunized with the C-terminus of hSSPN were assigned an internal identification number (289) by the company producer (ABclonal), while a cohort immunized with the LEL fragment were designated with 290 as an identification number. The first immune bleed (bleed I) was collected after two boosting injections on day 43. Bleeds II and III were collected on days 57 and 70. To evaluate the immune response in mice after the immunization, the mouse sera were tested with an ELISA against the biotin-conjugated fragments of hSSPN from the C-terminus (aa 219–243) and LEL fragment (aa 167–186). After the second boosting injection, mouse #289-02 exhibited the highest antibody titer (up to 3 × 10^−6^) to the mSSPN C-terminus in the ELISA (bleed I, Figure 5A). After the third boosting injection, two mice (#289-02 and #289-03) exhibited a high immune response to mSSPN with an antibody titer up to 10^−6^ (bleeds II and III; Supplementary Figure S4A,C). Of the mice immunized by the LEL fragment of hSSPN, mouse #290-01 developed the most robust response to the immunogen after the second booster (bleed I; Figure 5B) with retention upon subsequent boosters (bleeds II and III; Supplementary Figure S4B,D).

To select a mouse for the monoclonal antibody development, we tested the immune sera from bleeds II and III by immunoblotting, as described above (Supplementary Figure S5). A specific signal with endogenous SSPN was observed in both groups of immunized mice only after the fourth booster (bleed III). Among the sera from the mice immunized with the C-terminus fragment of hSSPN, we observed a specific signal with hSSPN in the immune sera from mouse #289-03 (one of the high responders in the ELISA) and mouse 289-01 (Supplementary Figure S5A, black arrows on the respective IB panels). Another top responder in the ELISA, the serum from mouse #289-02, did not recognize the endogenous mSSPN in the immunoblotting. Based on the results of the immunoblotting and IFA, mouse #289-03 was chosen for the monoclonal antibody development as the immune serum from this mouse generated the most robust signal with hSSPN in the immunoblotting (mouse #289-01 was used as a backup).

Among the mice immunized by the LEL fragment of hSSPN, we observed specific signals with both mSSPN (chevron on the respective IB panel; Supplementary Figure S5B) and hSSPN (black arrow) in the immune sera from only one mouse: #290-02. Interestingly, the immune serum from mouse 290-01, which demonstrated the highest antibody titers in the ELISA with recombinant hSSPN peptide (Supplementary Figure S4B,D), did not generate a specific signal to native human or mouse SSPN in the immunoblots (Supplementary Figure S5B). Mouse #290-02 was selected for the monoclonal antibody development as it recognized both mSSPN and hSSPN in the immunoblots (Supplementary Figure S5).

**Figure 6:**
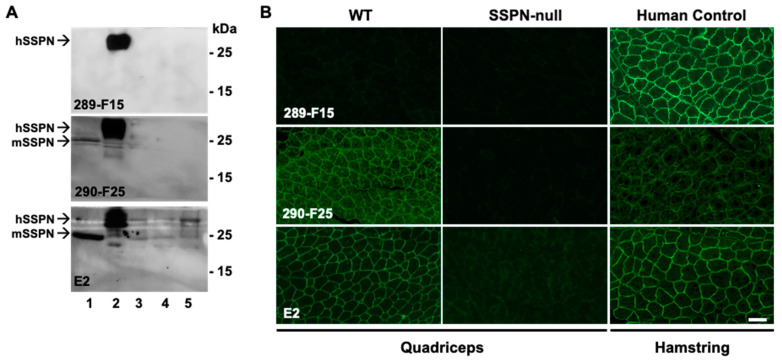
Validation of intermediate mouse hybridoma clones by immunoblotting and IFA. (**A**) mSSPN-TG (lane 1), hSSPN-TG (lane 2), WT (lane 3), *mdx* (lane 4), and SSPN-null (lane 5) with intermediate mouse clones 289-F15 and 290-F25 and commercially available mouse monoclonal antibody E2 (Santa Cruz). (There was 20 μg of total protein per line for all lines, except mSSPN where 2 μg of total protein per line was loaded. Film exposure time for all antibodies—2 min. (**B**) Immunofluorescence images of transverse cross-sections of the quadriceps muscle of the WT and SSPN-null mice and the human control (hamstring) stained with intermediate mouse clones 289-F15 and 290-F25 and mouse monoclonal antibody E2 (Santa Cruz). The acquisition time for all antibodies was 1 s. Scale bar, 50 μm.

After two additional boosters, splenocytes from the selected mice were harvested and fused with myeloma cells. After the fusion of the splenocytes from two mice with SP2/0-Ag14 myeloma cells, 2976 hybridoma clones were generated. The initial screening was performed by ABclonal with an ELISA with biotinylated synthetic peptides corresponding to the C-terminus and LEL fragment of hSSPN. One hundred clones (fifty per antigen) exhibiting the highest titers in the ELISA (Supplementary Tables S5 and S6) were selected for further evaluation by immunoblotting and IFA to determine the hybridomas for subcloning and expansion. Out of fifty clones against hSSPN C-terminus, 28 clones were active in recognizing hSSPN in the immunoblotting and IFA or in the IFA alone (Supplementary Table S7). Out of fifty tested clones against the hSSPN LEL fragments, 22 clones recognized mSSPN alone or both mSSPN and hSSPN in the immunoblotting and/or IFA. We selected twenty-three clones (marked with an asterisk in Supplementary Table S7) generating strong specific signals with endogenous SSPN for the subcloning and stabilization: fourteen from the group specific to the C-terminus of hSSPN and nine clones specific to the LEL. The immunoblotting of the muscle lysates prepared from all five mice genotypes (Table 1) and the IFA analysis of muscle from the murine and human samples revealed robust and specific SSPN detection from two representative intermediate clones: 289-F15 and 290-F25 (Figure 6). Side-by-side experiments were performed under identical conditions with mouse mAb E2 (Santa Cruz, SC-393187) to compare the signal intensity. Clone 289-F15 was developed against the C-terminus of hSSPN and recognized hSSPN in the immunoblotting and IFA. This clone exceeded E2 in terms of specificity (no extra bands in the immunoblot (Figure 6A) and sensitivity (a higher signal in the IFA with human muscle tissue compared to E2 after the same exposure time (Figure 6B). Clone 290-F25 recognized both the mouse and human SSPN in the immunoblotting and IFA and produced a high specific signal with hSSPN in the immunoblotting; however, the signal in the IFA with human muscle tissue was relatively low (Figure 6A,B).

After completion of the subcloning and stabilization, forty hybridoma cell supernatants (twenty clones per antigen) were selected based on the ELISA data (Supplementary Table S8) for re-screening in the immunoblotting and IFA to choose the 2–3 best clones per antigen as the final deliverables.

Evaluating the clones after subcloning and stabilization revealed that clones recognized endogenous SSPN only in the IFA. Neither clone generated a signal in the immunoblotting. This suggests that the clones working in both applications (including clones 289-F15 and 290-F25 (Figure 6)) were unstable and did not survive several rounds of subcloning. We selected five clones from the cohort generated against the LEL fragment and two clones specific to the C-terminus. The C-terminal clones were human-specific and clones against the LEL fragments were either mouse-specific or recognized both mouse and human SSPN (Table 3).

The monoclonal antibodies from selected clones were purified and evaluated by an indirect ELISA with biotinylated SSPN fragments. Eight serial dilutions, from 1 μg/mL to 7.8 ng/mL of antibodies, were analyzed, and all antibodies recognized SSPN in the ELISA at all tested concentrations (Supplementary Table S9A,B).

**Figure 7:**
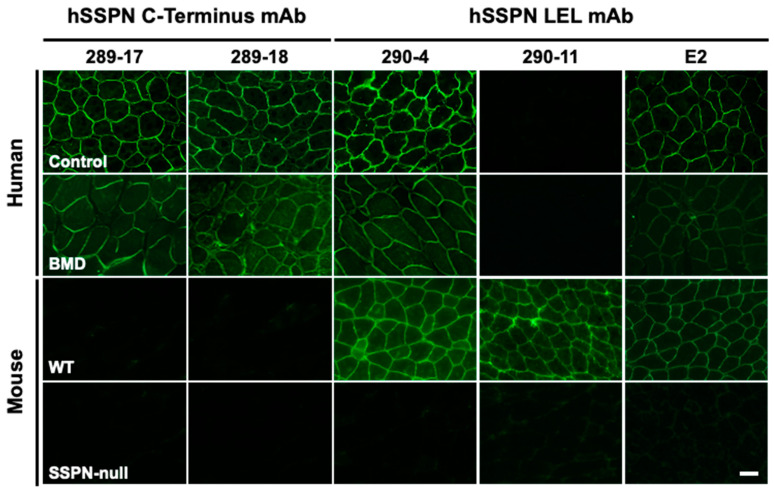
Validation of final mouse monoclonal antibodies to C-terminus and LEL fragment of human SSPN by IFA. Immunofluorescence images of transverse cross-sections of human healthy control, human Becker muscular dystrophy (BMD) skeletal muscles, and mouse WT and mouse SSPN-null quadriceps stained with newly developed mouse monoclonal antibodies to hSSPN C-terminus (clones 289-17, 289-18), hSSPN LEL fragment (clones 290-4, 290-11), and commercially available mouse monoclonal antibody E2 for comparison. Newly developed mouse monoclonal antibodies to the hSSPN C-terminus are human-specific and do not cross-react with mouse SSPN. Clone 290-04, developed to the hSSPN LEL fragment, recognizes both mSSPN and hSSPN, and clone 290-11 is mouse-specific. All three human-specific clones shown in the figure recognize hSSPN in muscles from the BMD patient more efficiently than E2. The acquisition time for all antibodies was 1 s. Scale bar, 50 μm.

An IFA evaluation of the selected clones demonstrated that the monoclonal antibodies recognizing endogenous mSSPN (Figure 7, clones 290-04 and 290-11) generate more intense signals with mSSPN in the muscle sections from wild-type mice compared to E2 monoclonal antibodies. The newly developed hSSPN-specific antibodies significantly overperform E2 and effectively recognize endogenous hSSPN in the muscle sections from the, control (healthy human) and from patients with Becker muscular dystrophy, where SSPN is reduced substantially at the sarcolemma (Figure 7, clones 289-17, 289-18, and 290-04). This makes our human-specific mouse monoclonal antibodies a unique tool for SSPN detection for research and clinical purposes.

**Figure 8:**
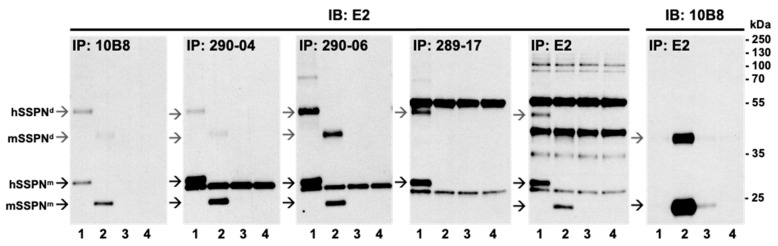
Affinity purification of SSPN by newly developed monoclonal antibodies. Quadricep muscle lysates from hSSPN-TG (lane 1), mSSPN-TG (lane 2), WT (lane 3), and SSPN-null (lane 4) were immunoprecipitated with the indicated rabbit (10B8) or mouse (290-04, 290-06, 289-17, and E2) monoclonal antibodies. Successful pull-down was assessed by immunoblotting equimolar amounts of lysates with E2 or 10B8 anti-SSPN antibodies. Lysates incubated with protein A/G PLUS-agarose beads without antibodies were used as a negative control (S.6). Black arrow: SSPN monomer (SSPN^m^); gray arrow: SSPN dimer (SSPN^d^); and IB: antibody used for immunoblotting. All blots were processed in parallel and developed with an identical film exposure time.

To interrogate whether our rabbit and mouse antibodies would affinity purify SSPN, we performed immunoprecipitation on the lysates from the quadriceps muscle of the wild-type, mSSPN-TG, hSSPN-TG, and SSPN-null mice utilizing the nine newly developed monoclonal antibodies and the commercially available E2. The efficiency of the SSPN pull-down was assessed by a SDS-PAGE analysis of the eluates followed by immunoblotting with either the E2 antibody, which recognizes both human and mouse SSPN, or the rabbit monoclonal antibody 10B8. All tested antibodies captured SSPN with variable affinity (Figure 8 and Supplementary Figure S6: black and gray arrows). In addition to the monomer (Figure 8 and Supplementary Figure S6: black arrows), we observed SSPN assembled as homo-oligomers (Figure 8 and Supplementary Figure S6: gray arrows), consistent with its function as a tetraspanin-like scaffolding protein. Although the E2 antibody successfully captured SSPN, the newly developed antibodies resulted in a higher specificity of affinity-purified SSPN with a markedly reduced background (Figure 8 and Supplementary Figure S6). When immunoblotting with E2, we were unable to detect endogenous SSPN in the WT eluates (Figure 8 and Supplementary Figure S6). However, when the lysates were affinity purified with E2 followed by analysis of the eluates with either E2 or 10B8 antibodies, we found that endogenous SSPN in the wild-type samples was detected by 10B8 (Figure 8). We reasoned that we were unable to observe endogenous SSPN after the affinity purification of the wild-type lysates due to the lower levels of endogenous SSPN coupled with the reduced sensitivity of E2 relative to our newly developed rabbit monoclonal 10B8 antibody. All newly developed rabbit monoclonal antibodies recognized only mouse SSPN in the IFA and immunoblotting (Figure 4 and Table 2). However, clone 10B8 was able to capture both mouse and human SSPN during the affinity purification (Figure 7). This clone captured mSSPN with a greater affinity than hSSPN (Figure 7 and Table 2). Another clone, 20E11, was only able to capture mSSPN (Supplementary Figure S6 and Table 2), suggesting that these rabbit monoclonal antibodies recognize distinct epitopes within the N-terminus of SSPN. The N-terminus peptide of mSSPN shares 52% identity with hSSPN (Figure 1D); however, the ability of clone 10B8 to capture hSSPN was unexpected, since it was unable to detect hSSPN by immunoblot (Figure 4A) or immunofluorescence analysis (Figure 4B). All newly produced antibodies targeting the LEL domain (290-04, 290-05, 290-17, 290-06, and 290-11) were generated using residues 167–186 of the hSSPN and detected both mouse and human SSPN in immunoprecipitation (Figure 8, Supplementary Figure S6, and Table 3). These antibodies could not detect SSPN by immunoblotting, suggesting that the epitope binds a fully or partially folded polypeptide that is likely denatured with the SDS treatment during immunoblotting. All of these clones captured hSSPN with a greater affinity relative to mSSPN (Figure 8 and Supplementary Figure S6), consistent with the use of human peptide, which shares 95% identity to mSSPN (Figure 1D). Taken together with the performance of these antibodies in the immunofluorescence analysis and ELISA, these data suggest that 290-11, 290-17, and 290-04/290-05 have distinct epitopes within the LEL region. Antibodies 289-17 and 289-18 were developed against residues 219–243 of the human C-terminus, which shares 64% identity with mSSPN (Figure 1D), and capture only hSSPN, consistent with our immunofluorescence analysis. Our newly developed antibodies bind to distinct epitopes within SSPN, in addition to selectively capturing either mouse or human SSPN or both, which will be beneficial in downstream applications.

## 3. Discussion

The dendrogram analysis shows SSPN has many structural similarities to the transmembrane four (TM4SF) or tetraspanin family of proteins [3]. The tetraspanin family of integral membrane proteins, consisting of 33 members [27], have emerged as *bona fide* organizers of microdomains in the plasma membrane [28,29,30]. The most distinctive feature that qualifies tetraspanins to organize larger protein complexes in the membrane is their ability to interact with other proteins [31,32]. The transmembrane domains of tetraspanin proteins undergo intramolecular interactions that support the functional protein conformation and mediate homophilic and heterophilic interactions [31,33]. SSPN contains four α-helical transmembrane (TM) regions (TM1–TM4), two extracellular loops (EL1 and EL2), and three cytoplasmic (CP) regions (N-terminus, C-terminus, and a short loop that connects TM2 and TM3) (Figure 1A,C). These topological domains contribute differently to the structure of SSPN and its ability to undergo intramolecular and intermolecular interactions [33]. SSPN possesses some, but not all, of the conserved Cys residues that define the core members of the tetraspanin superfamily [34]. However, like tetraspanins, SSPN forms oligomers [32] and interacts with other proteins [33] at the plasma membrane. The EL2 of SSPN is essential for its targeting of the sarcolemma [6] and SSPN oligomerization [35], and it is involved in heterophilic interactions with other proteins [36]. EL2 forms the primary binding site for the sarcoglycans, and the thiol bonds between the SSPN and sarcoglycans may be one mechanism by which the SSPN–sarcoglycan subcomplex maintains its integrity in the membrane [6]. The cytoplasmic regions of some tetraspanin proteins have been found to interact with cytosolic signaling or scaffolding proteins [37,38]. In the case of SSPN, intracellular N- and C-termini contribute to the stability of SSPN-mediated webs [6].

High-titer-specific antibodies to SSPN are in high demand in the basic research of protein–protein interactions within adhesion complexes and for evaluating protein replacement therapies of muscular dystrophies [39]. The discovery of antibodies against multi-pass transmembrane proteins is challenged by the complications with target presentation, such as poor expression, low solubility, and unnatural conformations that are observed within recombinantly produced material [40]. In the case of SSPN, antibody development possesses a great challenge due to the limited antigenic repertoire of this protein because of its small size and primarily membrane localization (Figure 1 and Supplementary Figure S1). The major limitation of currently available commercial antibodies is poor specificity causing a high background in various applications including immunoblotting and IFA. For instance, mouse monoclonal antibody E2 produces extra bands in immunoblotting interfering with the SSPN-specific signal (Figure 6A). Specific and robust antibodies are vital for the evaluation of the efficiency of SSPN-based therapy in mouse models of DMD, *mdx* mice. SSPN is reduced in the muscle cell membranes of *mdx* mice, making detecting this protein extremely challenging. To our knowledge, no currently commercially available anti-SSPN antibodies reliably detect the variations in protein expression in the context of low SSPN abundance at the sarcolemma. To address the barriers with commercially available antibodies, we created and validated a panel of polyclonal and monoclonal antibodies to SSPN of mouse and human origin. To expand a range of applications, we developed rabbit and mouse antibodies against three distinct epitopes of the SSPN protein: N-terminus, large extracellular loop, and C-terminus. Our antibodies recognize human and mouse SSPN and create a unique tool for detecting this protein in patients with muscular dystrophies and the murine models of muscle diseases.

Our rabbit antibodies recognize the N-terminus fragment of mSSPN. We chose a polypeptide fragment consisting of the first 25 amino acids of mSSPN based on previous experience of the successful creation of robust, high-titer rabbit polyclonal antibodies to SSPN [4,8,12,13]. Based on 52% amino acid identity between mouse and human SSPN N-terminus, we expected new rabbit antibodies to be mouse-specific. The whole panel of rabbit polyclonal and monoclonal antibodies bind mouse SSPN and do not cross-react with hSSPN in immunoblotting and IFA (Table 1). These antibodies tested positively in various immunoassays, such as immunoblotting, IFA, and an ELISA, generating specific and robust signals in all applications (Figure 3 and Figure 4).

**Figure 9:**
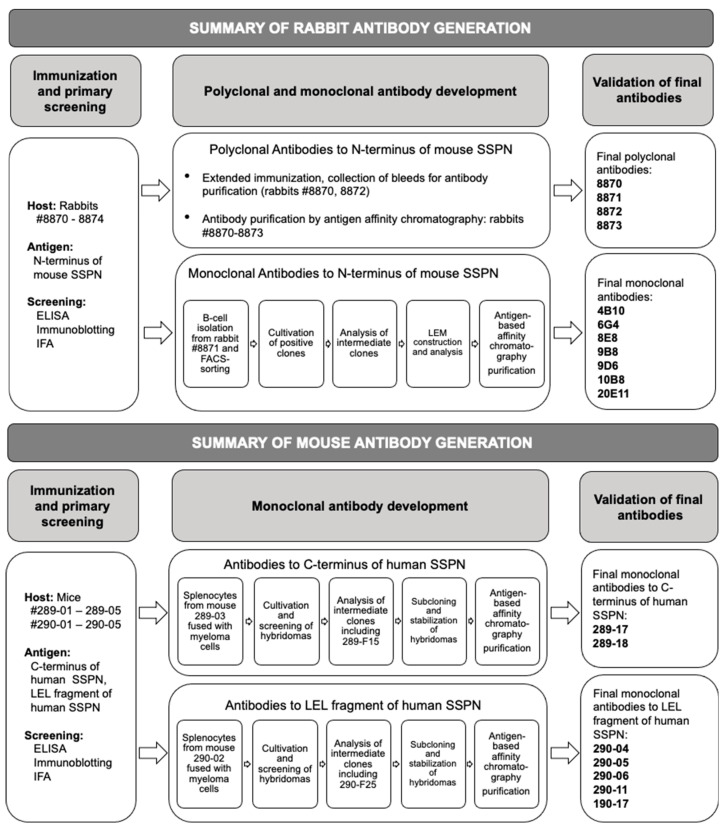
Summary of development of rabbit and mouse antibodies to SSPN of mouse and human origin.

The SSPN protein exhibits potential immunogenic epitopes within its large extracellular loop and the N- and C-termini. We used the commercially available mouse monoclonal antibody E2 as a reference antibody. According to the manufacturer, this antibody developed against aa 162–199 of hSSPN (Figure 1A,B). We chose fragment aa 167–186 based on our previous evaluation of the immunogenicity of SSPN fragments and predicted B-cell linear epitopes (Supplementary Figure S1). The results of the generation of mouse monoclonal antibodies to small hydrophobic proteins can be unpredictable. To increase the chances of obtaining mouse antibodies to SSPN, we included an additional potentially immunogenic region from the C-terminus of hSSPN and generated antibodies against two fragments: LEL and C-terminus. Based on the amino acid identity of the LEL fragment between the mouse and human SSPN (95%), we expected our antibodies to cross-react with the SSPN from mice and humans. The antibodies to the C-terminus were expected to be human-specific based on the fragment’s 64% amino acid identity between mice and humans. After validation of our mouse monoclonal antibodies, we found that the C-terminal clones are human-specific, as expected. Among the LEL-specific clones, we observed clones recognizing both mouse and human SSPN and, unexpectedly, two mouse-specific clones. Another interesting observation is that none of the intermediate clones detecting endogenous SSPN in IB and IFA (Figure 6) survived the subcloning steps. A summary of the antibody development is presented in Figure 9. The final mouse clones produce robust signals in the IFA but not in immunoblotting. Our human-specific monoclonal antibodies recognize endogenous SSPN in muscles from patients with Becker muscular dystrophy (BMD), where an abundance of this protein is significantly reduced compared to the levels of the SSPN protein at the sarcolemma of healthy muscles [9,11,41]. These data support the idea that the antibody reagents will be excellent tools for basic and translational research and open doors for the potential use of SSPN as a biomarker and therapeutic assessment for Duchenne and limb–girdle muscular dystrophies. Tetraspanin proteins have emerged as critical players in malignancy [42]. SSPN splicing is disrupted in many lung tumors [43], and the differential expression of SSPN has been reported in renal cell carcinoma [44]. These facts suggest the potential expansion of SSPN antibody usage beyond the field of muscular dystrophies. They have potential applications in a variety of conditions, including metabolic disorders, infectious diseases, and cancer cachexia.

## 4. Materials and Methods

### 4.1. SSPN B-Cell Linear Epitope Prediction

To identify possible B-cell epitopes within the SSPN protein, we used the Bepipred Linear Epitope Prediction 2.0 tool from IEDB Analysis Resource (http://tools.iedb.org/) [45], accessed on 15 February 2020, as well as findings from the prior studies reporting generation of robust SSPN antibodies [4,13,18,27,45]. We analyzed mouse SSPN (mSSPN; GenBank accession number U02487) and human SSPN (hSSPN; GenBank accession number AF016028) polypeptides to identify the most immunogenic fragments for antibody generation (Supplementary Figure S1).

### 4.2. Rabbit Antibody Development to N-Terminus of Mouse SSPN

#### 4.2.1. Recombinant SSPN Protein Production

We generated two recombinant SSPN fusion proteins for rabbit immunizations, as described below.

An N-terminal fragment of mSSPN protein (amino acids (aa) 1–25, MGRKPSPRAQELPEEEARTCCGCRF, U02487) was fused with a glutathione S-transferase (GST) tag. The GST fusion to the N-terminus of the mouse SSPN (GST-NT) was engineered by subcloning a cDNA fragment encoding the first 25 amino acids of mSSPN into the BamHI and EcoRI sites of the pGEX4T1 vector (Amersham Biosciences, Amersham, UK) [6,8,12]. The GST–SSPN fusion protein was used as an antigen for the antibody production. The GST–SSPN fusion protein was expressed and purified from *E. coli* Rosetta (DE3) by ABclonal Technology (Massachusetts, IL, USA). In brief, bacterial cells were grown in an LB medium at 37 °C until they reached a cellular optimal density (OD_600_) range of 0.5 to 0.6. At this point, the recombinant protein expression was induced during exponential growth by treatment with 0.4 mM isopropyl-1-thio-β-D-galactopyranoside (IPTG) at 37 °C for four hours. The cells were harvested and lysed by sonication in PBS plus 1% Triton X-100. The clarified lysates were affinity purified using glutathione–sepharose column chromatography according to the manufacturer’s instructions (ThermoFisher Scientific, 16109, Waltham, MA, USA). After the purification, 10–12 mg of the GST-fused SSPN protein per 300 mL of growth medium was obtained.

The N-terminal fragment of mSSPN protein (aa 1–25) was fused with a small ubiquitin-like modifier (SUMO) tag. This recombinant fusion protein was generated by ABclonal by first subcloning the cDNA fragment encoding the first 25 amino acids of mSSPN into the pET-28a SUMO expression vector. The SUMO-tagged SSPN was used for antibody screening purposes. The protein expression in the *E. coli* Rosetta (DE3) strain was induced with 0.4 mM IPTG for four hours at 37 °C. The cells were harvested and lysed by sonication in PBS plus 1% Triton X-100. The protein purification was performed by ABclonal using immobilized nickel ion affinity column chromatography (ThermoFisher Scientific, 90098). On average, 8–10 mg of SUMO-fused SSPN protein per 300 mL of growth medium was obtained after the purification.

#### 4.2.2. Rabbit Immunization

Five adult New Zealand white rabbits (females) were immunized with the purified GST recombinant fusion proteins described above to generate the rabbit polyclonal and monoclonal antibodies. The rabbits were injected intramuscularly (in both quadriceps) and subcutaneously with 1 mg (total) of purified N-terminal SSPN GST-fused recombinant protein in Freund’s Complete Adjuvant using previously established immunization protocols that have been shown to generate robust, high-titer antibodies against SSPN [4,8,12,13]. Before the immunization, pre-immune sera (5 mL from each rabbit) were collected from the lateral ear vein. After the initial immunization, each rabbit received three subcutaneous boosters (days 7, 21, and 42) with 500 μg of antigen in Freund’s Incomplete Adjuvant (Supplementary Table S1). Bleeds I and II were collected from the lateral ear vein after the second and third boosters (days 28 and 49) to test the immune response to SSPN protein. The rabbits selected for the generation of monoclonal antibodies received a final injection with 500 μg of antigen intravenously four days before the spleen collection (Supplementary Table S1). The rabbits selected for the extended immunization and generation of polyclonal antibodies received four additional boosters with 250 μg of antigen in Freund’s Incomplete Adjuvant via the subcutaneous route of administration (two posterior sites). Production bleeds III–V were collected from the lateral ear vein seven days after each booster (Supplementary Table S2). The terminal bleeds for the polyclonal antibody generation were performed via cardiac puncture under anesthesia. ABclonal performed the animal housing, immunization, and blood collection procedures according to the approved protocols and guidelines of the Institutional Animal Care and Use Committee (IACUC).

#### 4.2.3. Indirect Enzyme-Linked Immunosorbent Assay (ELISA) for Detection of Recombinant SSPN

To confirm whether the rabbits developed an immune response to SSPN, the sera from bleeds I and II were analyzed by ABclonal using an indirect ELISA with the purified SUMO-tagged recombinant SSPN protein (described above). Overnight, 96-well plates were precoated with a 25 μL/well of SUMO-tagged SSPN (0.5 µg/mL) in a carbonate–bicarbonate coating buffer at a pH of 9.66 (Sigma, SRE0102, Burlington, MA, USA). After three rounds of washing with the wash buffer (a sodium phosphate buffer at a pH of 7.4 (ThermoFisher Scientific, J62152-K2) with 0.1% Tween-20), the plates were blocked with 3% BSA in a sodium phosphate buffer at a pH of 7.4 for two hours, and then probed with serial dilutions of antisera in a carbonate–bicarbonate coating buffer at a pH of 9.66 and incubated for one hour at 37 °C. After another three rounds of washing, a peroxidase-conjugated goat anti-rabbit IgG antibody (Jackson ImmunoResearch, 111-005-046, West Grove, PA, USA) at 1:5000 in a dilution buffer (1% BSA in a sodium phosphate buffer at a pH of 7.4 with 0.1% Tween-20) was added to the plate and incubated for one hour at RT. The plate was washed four times, as described above. The enzymatic reaction was conducted with 100 µL/well in a ready-to-use tetramethylbenzidine (TMB) solution (ThermoFisher Scientific, N301) at RT and stopped with a 50 µL/well of 2 M sulfuric acid. The optical density (OD) was measured at 450 nm using a SpectraMax M2 microplate reader (Molecular Devices, San Jose, CA, USA). The antibody titer was estimated using endpoint titration; the original sera were first diluted at 1:1000, followed by 8-point 1:3 serial dilutions. Each dilution was run in duplicate. Prism software (GraphPad Prism version 10.2.3) was used to plot the data and perform the statistical analysis. The average absorbance values for each set of duplicate negative controls (the pre-immune sera from all five rabbits combined) and experimental samples were calculated. The ELISA background signal was determined as an average of the negative samples with three standard deviations. The highest dilution of immune sera that generated an OD_450_ twice exceeding the background was considered as the antibody titer.

#### 4.2.4. Immunoblotting for Detection of SSPN in Skeletal Muscle Protein Lysates

We analyzed the rabbit sera by an immunoblotting of the total muscle lysates from quadriceps of wild-type (C57BL6/J) (Jackson Laboratories, Bar Harbor, ME, USA), *mdx* (C57BL/10ScSn-Dmd *mdx*/J) (Jackson Laboratories), mouse SSPN-TG (transgenic line 28, Ln28) [17], human SSPN-TG (transgenic line 3, Ln3) [10], and SSPN-null mice (Table 1) [23]. The mice were maintained following the guidelines established by the Institutional Animal Care and Use Committee at the University of California, Los Angeles, and approval for the mice in this study was granted by the UCLA Institutional Animals Care and Use Committee (IACUC) (protocol #2000-029-61G). The quadriceps muscles were harvested from 12–15-weeks-old male mice of each genotype, snap-frozen in liquid nitrogen, pulverized under liquid nitrogen, and homogenized on ice in a 30× volume of RIPA buffer containing 50 mM of Tris-HCl at a pH of 7.4, 150 mM of NaCl, 1% Triton X-100, 0.5% Na deoxycholate (DOC), and 0.1% sodium dodecyl sulfate (SDS), with a protease inhibitor cocktail (Thermo Fisher Scientific, 1860932) and a phosphatase inhibitor cocktail (Thermo Fisher Scientific, 78428). The lysates were incubated for one hour at 4 °C with rotation and clarified by centrifugation at 18,000× *g* for 15 min. The protein concentrations of the clarified supernatants were quantified using the DC Protein Assay (Bio-Rad, 5000114, Hercules, CA, USA). The supernatants were separated by SDS electrophoresis (20 μg of total protein) on 12% Bolt Bis-Tris gels (Thermo Fisher Scientific, NW00122) in MOPS SDS Running Buffer (Invitrogen, B000102, Waltham, MA, USA). The PageRuler Plus Prestained Protein Ladder (Thermo Fisher Scientific, 26619) was used as a molecular weight marker. The immunoblotting was performed as previously described [15]. Briefly, nitrocellulose membranes (Li-Cor Biosciences, 926-31092, Lincoln, NE, USA) were blocked with a blocking buffer (5% nonfat dry milk (Nestle Carnation, Nestle, Konolfingen, Switzerland) in Tris-buffer at a pH of 7.4 (Thermo Fisher Scientific, 28379) with 0.1% Tween-20 (TBS-T)) followed by incubation with primary antibodies (rabbit Bs, purified polyclonal antibodies or supernatants from rabbit B-cell clones) overnight at 4 °C with gentle rotation. For the testing, rabbit bleeds I and II were diluted in the blocking buffer at 1:150 and 1:600, respectively (Figure 2), and the purified polyclonal and monoclonal antibodies were tested at 0.5–1 µg/mL concentrations (Figure 3B and Figure 4A). The membranes were washed three times, 5 min each, in TBS-T before applying the secondary antibody: horseradish peroxidase-conjugated anti-mouse (Abcam, Cambridge, UK, ab6708) or anti-rabbit IgG (Abcam, ab6721) 1:30,000 in the blocking buffer. The signal was detected with a SuperSignal West Pico Plus Chemiluminescence kit (Thermo Fisher Scientific, Waltham, MA, USA, 34580) on Blue Basic Autoradiography Film (GeneMate, F-9023-8X10).

#### 4.2.5. Indirect Immunofluorescent Analysis (IFA) for Detection of SSPN in Transverse Cryosections of Human and Mouse Skeletal Muscle

The human muscle control tissue was obtained from the left hamstring of a healthy 13-year-old male (protocol #21-000350) from anterior cruciate ligament (ACL) reconstruction, frozen in VWR histology cassettes (18000-240), and subsequently sectioned at 6 μm. The Becker muscular dystrophy (BMD) muscle tissue was obtained from open or core needle muscle biopsies from the biceps brachii or vastus lateralis from ambulatory male patients diagnosed with BMD. This study was approved by the Institutional Review Board at Cedars-Sinai Medical Center (protocols #27634, #33300), is in accord with the Declaration of Helsinki principles, and each subject gave informed written consent to participate. The human BMD muscle tissues were frozen in liquid nitrogen-cooled isopentane, mounted on Tissue Plus OCT (Fisher HealthCare, 4585, Waltham, MA, USA), and stored at −80 °C before being sectioned at 6 μm. The mouse quadriceps tissues from the WT and SSPN-null mice were frozen in liquid nitrogen-cooled isopentane, mounted on Tissue Plus OCT, and stored at −80 °C before cryosectioning at a thickness of 10 μm. Transverse cryosections on Superfrost Plus microscope slides (Thermo Fisher Scientific, 12-550-15) were blocked with 3% BSA in PBS at room temperature (RT) for 1 h, followed by incubation with an avidin/biotin blocking kit according to the manufacturer’s protocol (Vector Laboratories, SP-2001, Newark, CA, USA). The sections were washed with PBS at a pH of 7.4 (Fisher BioReagents, BP399-4, Waltham, MA, USA) and incubated with primary antibodies in MOM diluent (Vector Laboratories, BMK-2202) overnight at 4 °C. The primary antibodies were detected using biotinylated anti-mouse antibodies (BA-9200) (1:250; Vector Labs) and biotinylated anti-rabbit antibodies (BA-1000) (1:250; Vector Labs). Fluorescein-conjugated (A-2001) (1:500 in 3% BSA-PBS; Vector Labs) avidin D was used to detect the biotinylated secondary antibodies. The slides were wet mounted in Vectashield (Vector Laboratories, H-1200) with or without DAPI (Vector Laboratories, H-1000) before the analysis by microscopy on a Zeiss Axio Observer 7 equipped with an excitation filter 470/40, an emission filter 525/50, and a Colibri 7 LED as the light source. The images were captured with a Hamamatsu ORCA-Flash 4.0 V3 digital complementary metal oxide semiconductor camera and either EC Plan-Neofluar 10×/0.30 Ph1 or Plan-Apochromat 20×/0.8 M27 objectives using identical exposure time for all analyzed samples.

#### 4.2.6. Isolation and Sorting of Rabbit B-Cells

The isolation and sorting of the rabbit immune cells were performed by ABclonal using their proprietary technology based on antigen-specific single B-cell sorting [25,26]. The antigen-binding cells from the rabbit spleen were isolated by fluorescent-activated cell sorting (FACS) using anti-rabbit IgG Alexa Fluor 488 (Abcam, ab150077) and anti-rabbit IgM Alexa Fluor 647 (Abcam, ab150191) fluorochrome conjugates, along with SSPN N-terminus fragment conjugated with BP Fluor 405 (BroadPharm, BP-25537, San Diego, CA, USA) for the identification of the antigen-specific B cells. The antigen-specific IgG^+^/IgM^−^ B cells were sorted, and a single cell was placed into individual wells of 96-well plates. All individual B-cell clones (1920 in total) were expanded for 12 days with the ABclonal proprietary cell culture system. All B-cell supernatants were screened in the ELISA for reactivity to the SSPN recombinant SUMO-tagged antigen by ABclonal. The B-cell supernatants from the top 96 positive clones with the highest reactivity in the ELISA were sent to UCLA and subjected to validation by immunoblotting and IFA. Based on the results of these three assays, the top fifteen clones were selected for the IgG gene isolation and generation of the linear expression modules.

#### 4.2.7. Generation of the Linear Expression Modules (LEMs)

The total RNA was purified from each preserved B-cell pellet for the selected clones by ABclonal. The isolation of the mRNA and the amplification of the cDNA fragments of IgG were performed by a reverse transcription polymerase chain reaction (RT-PCR), as described previously [46]. Variable regions of heavy and light chains were amplified using cDNA after the reverse transcription and used for the construction of the LEMs [47] by ABclonal. The LEM construction was performed according to the companies’ protocol and involved inserting the Ig heavy chain and light chain genes into a linear construct for the transient transfection into mammalian cells. This approach facilitated higher throughput screening of the clones (due to a reduced need for subcloning relative to the methods that rely on generating expression vectors) [26].

The HEK293-T cells were transiently transfected with LEM constructs containing the coding sequences of the heavy chain and light chain fragments originating from the same sorted cells by ABclonal using their established protocols. The supernatants were harvested after three days for evaluation with an ELISA (Section 4.2.3), immunoblotting (Section 4.2.4), and IFA (Section 4.2.5) to choose several of the best clones for the small-scale rabbit recombinant antibody production.

### 4.3. Development of Mouse Antibodies to the Large Extracellular Loop and C-Terminus of Human SSPN

#### 4.3.1. Generation of Synthetic SSPN Polypeptides

Two peptides of hSSPN (GenBank accession number AF016028), one from the C-terminal region (aa 219–243, HRYQVFYVGVRICSLTASEGPQQKI) and a fragment of the large extracellular loop (LEL) (aa 167–186, PSSEPLSRTFVYRDVTDCTSC), were synthesized by ABclonal using the standard methods. To enhance the immunogenicity, the synthetic peptides were coupled to keyhole limpet hemocyanin (KLH) with the cross-linking reagent succinimidyl 4-(*N*-maleimidomethyl)cyclohexane 1-carboxylate (Thermo Fisher Scientific, 23456).

#### 4.3.2. Immunizations and Evaluation of Mouse Immune Response to hSSPN

ABclonal performed all of the housing and immunization procedures according to the approved protocols and guidelines of IACUC. Briefly, two-month-old female BALB/c mice (five mice per antigen) were injected subcutaneously with 100 μg of synthetic antigen peptide supplemented with Complete Freund’s Adjuvant for the primary injection (day 1) and 100 μg of antigen peptide supplemented with Incomplete Freund’s Adjuvant for the subsequent four boosting injections (days 22, 36, 50, and 64) (Supplemental Table S4). The first immune bleed (bleed I) was collected after two boosting injections on day 43. Bleeds II and III were collected on days 57 and 70, respectively.

To evaluate the immune response in mice after immunization, the mouse sera were tested by the ELISA against SSPN synthetic peptides by ABclonal. To analyze the immune response to the hSSPN C-terminal fragment, 96-well plates were precoated with biotin-conjugated hSSPN (aa 219–243, HRYQVFYVGVRICSLTASEGPQQKI, AF016028) at 1 µg/mL in a carbonate–bicarbonate coating buffer at a pH of 9.66 (Sigma, SRE0102) overnight. To analyze the immune response the to human SSPN LEL fragment, biotin-hSSPN (aa 167–186, PSSEPLSRTFVYRDVTDCTS, AF016028) at 1 µg/mL in a carbonate–bicarbonate coating buffer was used as an antigen. The mouse sera from bleeds II and III were first diluted at 1:1000, followed by 8-point 1:3 serial dilutions in a carbonate–bicarbonate coating buffer at a pH of 9.66, and incubated for 1 h at 37 °C. Peroxidase-conjugated AffiniPure Goat Anti-Mouse IgG (H + L) (Jackson ImmunoResearch, 111-005-046, West Grove, PA, USA), 1:10,000 in the dilution buffer (1% BSA in a sodium phosphate buffer at a pH of 7.4 with 0.1% Tween-20) was added to the plate and incubated for one hour at RT. All washing, blocking steps, enzymatic reaction, and antibody titer estimation were performed as described in Section 4.2.3. Bleeds II and III were tested in immunoblotting by ABclonal using the workflow described in Section 4.2.3 to choose the best mouse for the monoclonal antibody development.

#### 4.3.3. Mouse Monoclonal Antibody Generation

ABclonal used the standard hybridoma generation approach [48] to create the mouse monoclonal antibodies. For the monoclonal antibody generation, 150 μg of antigen without adjuvant was administered through peritoneal and intravenous (tail vein) routes three days before the splenectomy. The isolated murine spleen cells were fused with SP2/0-Ag14 cell line (ATCC, CRL-1581TM) myeloma cells using polyethylene glycol PEG1500 (Roche Diagnostics, 10783641001, Rotkreuz, Switzerland). To enhance the cloning efficiency and prevent the loss of high-producing clones, a methylcellulose-based semisolid medium was chosen for the hybridoma selection and cloning [49] where the fused cell suspension was mixed with a medium containing methylcellulose (Sigma, M7027) and plated in 100 mm Petri dishes (Thermo Fisher Scientific, 172931). After 10 to 14 days, single colonies visible to the naked eye were individually picked from the semisolid media and transferred into single wells of 96-well culture plates containing 200 µL of ClonaCell-HY medium E (Stemcell, 03805, Vancouver, Canada). A new sterile tip was used for each colony to maintain the clonality. The mini-pools were cultured for two days, and the culture supernatants were taken for analysis with an ELISA against hSSPN C-terminus and LEL biotinylated synthetic peptides, as described in Section 4.3.2, to detect the clones generating antibodies with the highest titer to hSSPN.

Based on the ELISA results, 100 clones (50 for each antigen; Supplementary Tables S5 and S6) were selected and sent to UCLA for testing by immunoblotting with the muscle lysates from wild-type, *mdx*, mouse SSPN-TG (mSSPN-TG), human SSPN-TG (hSSPN-TG), and SSPN-null mice using the workflow described in Section 4.2.4 and by immunofluorescence analysis with cryosections from WT and SSPN-null mice and human control tissue (Section 4.2.5). We analyzed the intermediate hybridoma clones for their ability to recognize endogenous SSPN with a high specificity (a strong specific signal with minimum background) in the immunoblotting and IFA (Supplementary Table S7) for the subcloning and expansion. Multiple rounds of subcloning were performed on selected clones until monoclonal hybridoma cell lines were established using a limiting dilution method [25] by ABclonal. After the subcloning and stabilization, the clones were evaluated using the ELISA by ABclonal, and 80 clones (40 per antigen) with the highest ELISA titers to SSPN (Supplementary Table S8A,B) were sent to UCLA for re-screening by immunoblotting and IFA. Based on the results of the re-screening, the seven clones that generated the strongest signals to endogenous SSPN with minimal background were selected as the final clones. These clones were re-tested with an ELISA (Supplementary Table S9A,B), and the mouse monoclonal antibodies were purified from selected clones by ABclonal using a HiTrap Protein A column (Cytiva, 17507901, Marlborough, MA, USA) and the ÄKTA pure protein purification system. The captured antibody was eluted with a Pierce IgG elution buffer (Thermo Fisher Scientific, 21004). The purified antibodies were pooled, concentrated, and buffer-exchanged to a PBS buffer (at a pH of 7.4) using the Amicon Ultra-15 (30 kDa) centrifugal filter (Millipore, UFC903024, Burlington, MA, USA). The purified antibodies were aliquoted and kept at 4 °C for short-term storage or −80 °C for long-term storage.

### 4.4. Determining the Efficiency of Immunoprecipitation for Affinity Purification of SSPN

The quadriceps muscles from WT (C57BL6/J) (Jackson Laboratories), mouse SSPN-TG (Ln28) [17], human SSPN-TG (Ln3) [10], and SSPN-null mice [23] (Table 1) were pulverized using a metal mortar and pestle. The ground tissue was Dounce homogenized in a 20× volume of ice-cold IP lysis buffer (50 mM of Tris, 150 mM of NaCl, and 1% NP-40 at a pH of 7.4) containing Halt protease (Thermo Fisher Scientific, 1860932) and phosphatase inhibitors (Thermo Scientific, 78428); transferred to microcentrifuge tubes; and rotated at 4 °C for one hour. The lysates were clarified by centrifuging at 20,000× *g* for 15 min at 4 °C. The protein concentration was determined with the DC Protein Assay (Bio-Rad, 5000113-115). The muscle lysates (250 mg) were pre-cleared to minimize non-specific binding using 50 μL of protein A/G PLUS-agarose beads (SCBT, sc2003) for one hour at 4 °C with gentle rotation. The beads were sedimented by centrifugation at 1000× *g* for 5 min at 4 °C, and the pre-cleared lysates were transferred to a new microcentrifuge tube containing 5 µg of antibodies followed by rotation for one hour at 4 °C. The SSPN-antibody conjugates were captured with 50 μL of protein A/G PLUS-agarose beads and pre-equilibrated with a cold IP wash buffer overnight at 4 °C with rotation, before washing five times with an IP wash buffer (50 mM of Tris, 150 mM of NaCl, and 0.1% NP-40 at a pH of 7.4) containing Halt protease and phosphatase inhibitors. The bound proteins were eluted by heating at 70 °C for 10 min with a 2× NuPAGE sample buffer (Thermo Fisher Scientific, NP0007). All tubes were pre-chilled on ice. The centrifugation steps were performed at 1000× *g* for 5 min at 4 °C. Equimolar amounts of eluates were resolved by SDS-PAGE under reducing conditions on 12% Bolt Bis-Tris gels (Thermo Fisher Scientific, NW00122BOX) and transferred onto nitrocellulose membrane (Licor Biosciences, Lincoln, NE, USA) at 100 V for two hours, as described in Section 4.2.4. The PageRuler Plus Prestained Protein Ladder (Thermo Scientific, PI26619, Waltham, MA, USA) was used as a molecular weight marker. The immunoblotting was performed as described in Section 4.2.4.

## 5. Conclusions

A unique panel of antibodies to murine and human SSPN were created as a resource for the research community working in the broad area of cell membrane biology.

The antibodies produced in two species, rabbits and mice, target three distinct SSPN epitopes and have an extended range of applications (Table 2 and Table 3).

The rabbit antibodies generated robust, specific signals, with the mouse SSPN outperforming the commercially available counterparts in various applications, including immunoblotting, IFA, and immunoprecipitation.

The rabbit and murine antibodies reliably detect SSPN in the low abundance typical of disease contexts, making them appropriate reagents for basic and translational research, as well as clinical applications.

## Figures and Tables

**Table 1:** Murine models used for SSPN antibody characterization.

Mouse Strain	Description	SSPN Protein Expression
Wild type	C57BL/6 murine strain	Normal baseline level of mSSPN
*mdx*	Dystrophin-deficient murine model of DMD	Low expression of mSSPN (5–10% of wild-type levels) [10]
mSSPN-TG	C57BL/6 mice with transgenic overexpression of murine SSPN in skeletal muscle	Very high level of mSSPN (~30× wild-type levels) [15]
hSSPN-TG	C57BL/6 mice with transgenic overexpression of human SSPN in skeletal muscle	Increased level of hSSPN (~3× wild-type levels) [10]
SSPN-null	C57BL/6 mice homozygous for a SSPN-null mutation from C57BL/6 background (negative control)	None [23]

**Table 2:** Summary of rabbit antibodies to sarcospan of mouse origin.

Antibody	Clonality	Epitope	Applications and Specificity			
			IFA	IB	ELISA	IP
8870	Polyclonal	Mouse SSPN aa 1-25 (N-terminus)	Mouse SSPN	Mouse SSPN	Mouse SSPN	Mouse SSPN
8871, 8872, 8873						NT *
10B8	Monoclonal					Mouse SSPN Human SSPN
20E11						Mouse SSPN
4B10, 6G4, 8E8, 9B8, 9D6						NT *

* NT—Not Tested.

**Table 3:** Summary of mouse monoclonal antibodies to sarcospan of human origin.

Antibody	Subclass of Monoclonal Antibody	Epitope	Applications and Specificity			
			IFA	IB	ELISA	IP
290-04, 290-05	IgG2A	Human SSPN aa 167-186 (LEL)	Human SSPN Mouse SSPN	ND **	Human SSPN Mouse SSPN	Human SSPN Mouse SSPN
290-17	IgG1					
290-06, 290-11	IgG2A		Mouse SSPN	ND **	Human SSPN	
289-17, 289-18	IgG1	Human SSPN aa 219–243 (C-terminus)	Human SSPN	ND **	Human SSPN	Human SSPN

** ND-Not Detected.

## Data Availability

Data is contained within the article and Supplementary Materials.

## Supplementary Materials

The following supporting information can be downloaded at https://www.mdpi.com/article/10.3390/ijms25116121/s1.

## Abbreviations

BMD—Becker muscular dystrophy, DG—dystroglycan, DGC—dystrophin–glycoprotein complex, DMD—Duchenne muscular dystrophy, ECM—extracellular matrix, GST—glutathione S-transferase, hSSPN—human sarcospan, hSSPN-TG—mice with transgenic overexpression of human sarcospan, IB—immunoblotting, ICC—immunocytochemistry, IHC—immunohistochemistry, IFA—immunofluorescence analysis, IP—immunoprecipitation, IPTG—isopropyl-1-thio-β-D-galactopyranoside, LEL—large extracellular loop, LEM—linear expression module, LGMD—limb–girdle muscular dystrophy, mSSPN—mouse sarcospan, mSSPN-TG—mice with transgenic overexpression of mouse sarcospan, SSPN—sarcospan, SUMO—small ubiquitin-like modifier, and UGC—utrophin–glycoprotein complex.
