# Supplementary material for: The Development of Robust Antibodies to Sarcospan, a Dystrophin- and Integrin-Associated Protein, for Basic and Translational Research
Source: Int J Mol Sci. 2024 Jun 1;25(11):6121. doi: 10.3390/ijms25116121 (PMC11173052; doi:10.3390/ijms25116121)
Supplement: Supplementary file 1 [file ijms-25-06121-s001.zip › ijms-2986773-supplementary.pptx]

## Slide 1
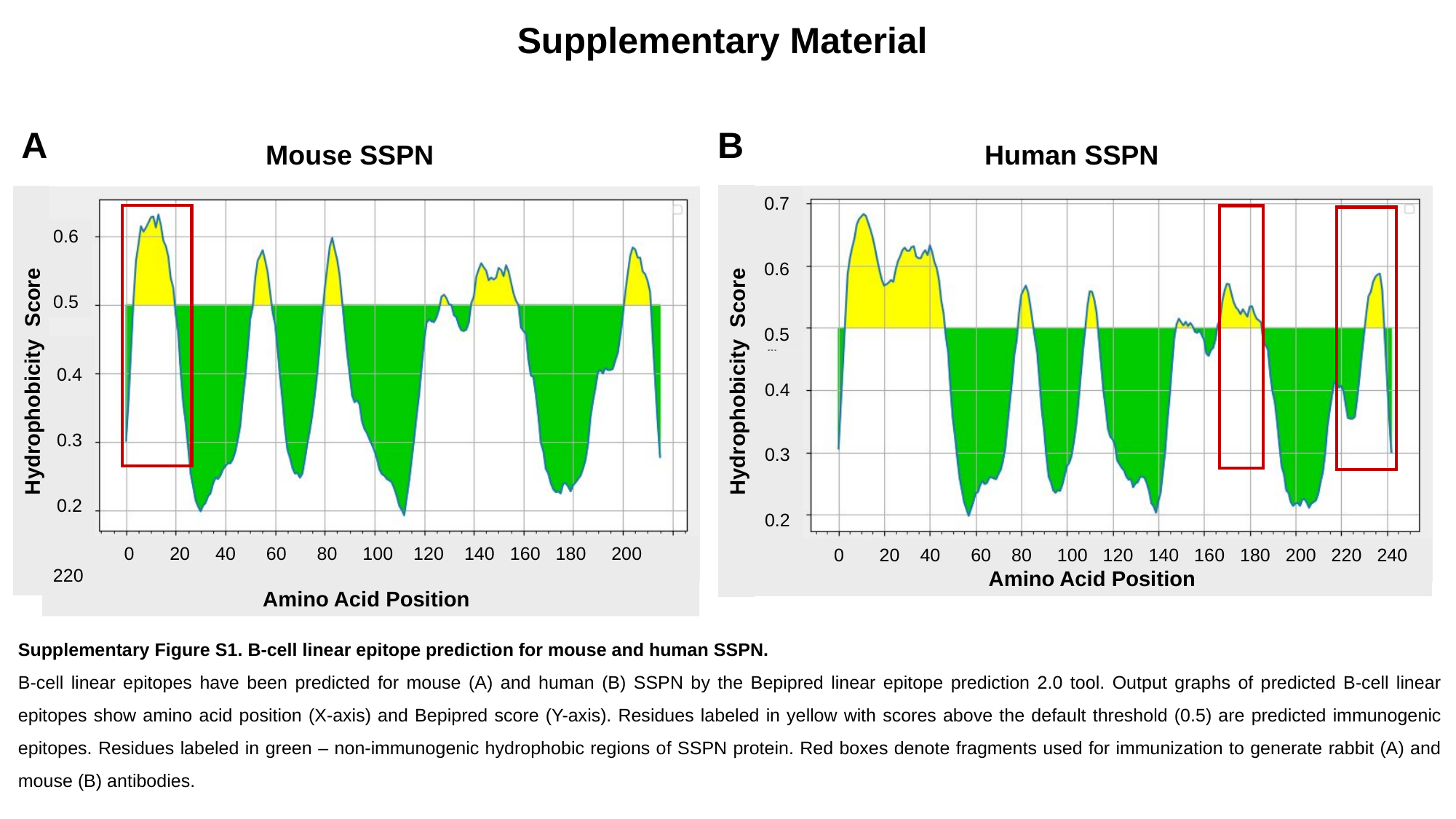

Supplementary Material
A
B
Mouse SSPN
Human SSPN
0.7
0.6
0.5
0.4
0.3
0.2
 Hydrophobicity Score
 0 20 40 60 80 100 120 140 160 180 200 220 240
 Amino Acid Position
0.6
0.5
0.4
0.3
0.2
 Hydrophobicity Score
 0 20 40 60 80 100 120 140 160 180 200 220
 Amino Acid Position
Supplementary Figure S1. B-cell linear epitope prediction for mouse and human SSPN.
B-cell linear epitopes have been predicted for mouse (A) and human (B) SSPN by the Bepipred linear epitope prediction 2.0 tool. Output graphs of predicted B-cell linear epitopes show amino acid position (X-axis) and Bepipred score (Y-axis). Residues labeled in yellow with scores above the default threshold (0.5) are predicted immunogenic epitopes. Residues labeled in green – non-immunogenic hydrophobic regions of SSPN protein. Red boxes denote fragments used for immunization to generate rabbit (A) and mouse (B) antibodies.

## Slide 2
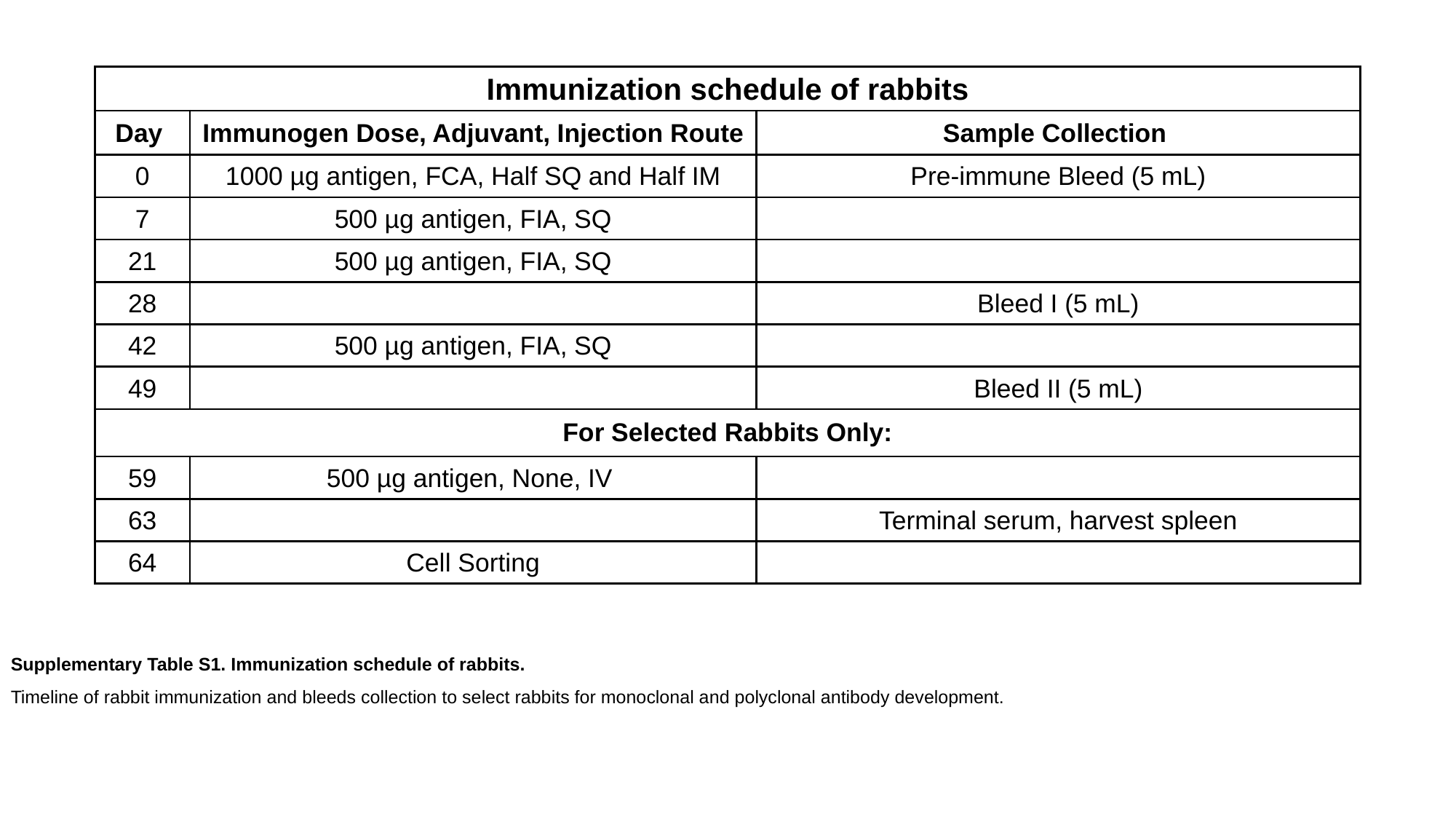

| Immunization schedule of rabbits | | |
| --- | --- | --- |
| Day | Immunogen Dose, Adjuvant, Injection Route | Sample Collection |
| 0 | 1000 µg antigen, FCA, Half SQ and Half IM | Pre-immune Bleed (5 mL) |
| 7 | 500 µg antigen, FIA, SQ | |
| 21 | 500 µg antigen, FIA, SQ | |
| 28 | | Bleed I (5 mL) |
| 42 | 500 µg antigen, FIA, SQ | |
| 49 | | Bleed II (5 mL) |
| For Selected Rabbits Only: | | |
| 59 | 500 µg antigen, None, IV | |
| 63 | | Terminal serum, harvest spleen |
| 64 | Cell Sorting | |
Supplementary Table S1. Immunization schedule of rabbits.
Timeline of rabbit immunization and bleeds collection to select rabbits for monoclonal and polyclonal antibody development.

## Slide 3
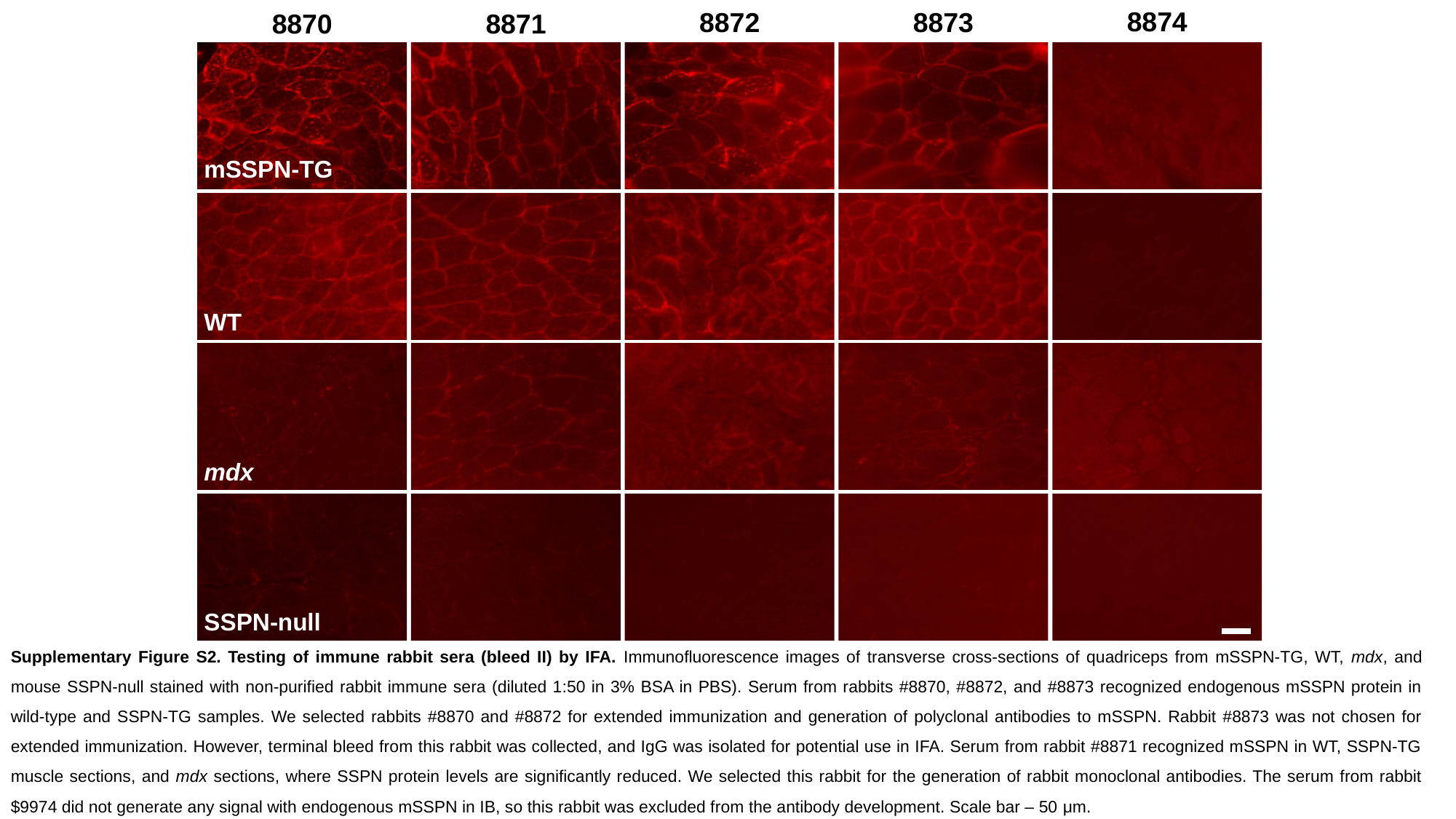

8874
8872
8873
8870
8871
mSSPN-TG
WT
mdx
SSPN-null
Supplementary Figure S2. Testing of immune rabbit sera (bleed II) by IFA. Immunofluorescence images of transverse cross-sections of quadriceps from mSSPN-TG, WT, mdx, and mouse SSPN-null stained with non-purified rabbit immune sera (diluted 1:50 in 3% BSA in PBS). Serum from rabbits #8870, #8872, and #8873 recognized endogenous mSSPN protein in wild-type and SSPN-TG samples. We selected rabbits #8870 and #8872 for extended immunization and generation of polyclonal antibodies to mSSPN. Rabbit #8873 was not chosen for extended immunization. However, terminal bleed from this rabbit was collected, and IgG was isolated for potential use in IFA. Serum from rabbit #8871 recognized mSSPN in WT, SSPN-TG muscle sections, and mdx sections, where SSPN protein levels are significantly reduced. We selected this rabbit for the generation of rabbit monoclonal antibodies. The serum from rabbit $9974 did not generate any signal with endogenous mSSPN in IB, so this rabbit was excluded from the antibody development. Scale bar – 50 μm.

## Slide 4
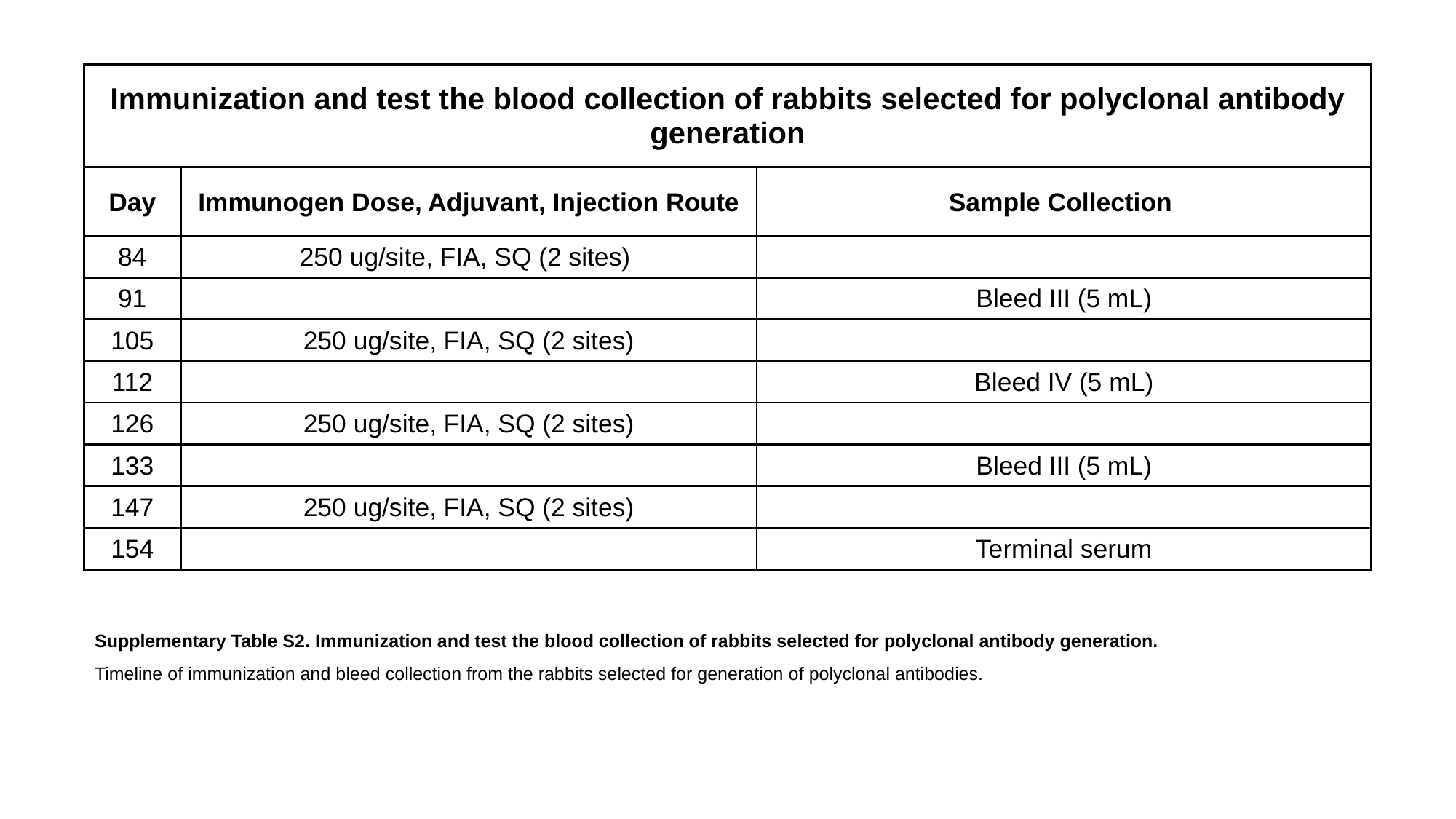

| Immunization and test the blood collection of rabbits selected for polyclonal antibody generation | | |
| --- | --- | --- |
| Day | Immunogen Dose, Adjuvant, Injection Route | Sample Collection |
| 84 | 250 ug/site, FIA, SQ (2 sites) | |
| 91 | | Bleed III (5 mL) |
| 105 | 250 ug/site, FIA, SQ (2 sites) | |
| 112 | | Bleed IV (5 mL) |
| 126 | 250 ug/site, FIA, SQ (2 sites) | |
| 133 | | Bleed III (5 mL) |
| 147 | 250 ug/site, FIA, SQ (2 sites) | |
| 154 | | Terminal serum |
Supplementary Table S2. Immunization and test the blood collection of rabbits selected for polyclonal antibody generation.
Timeline of immunization and bleed collection from the rabbits selected for generation of polyclonal antibodies.

## Slide 5
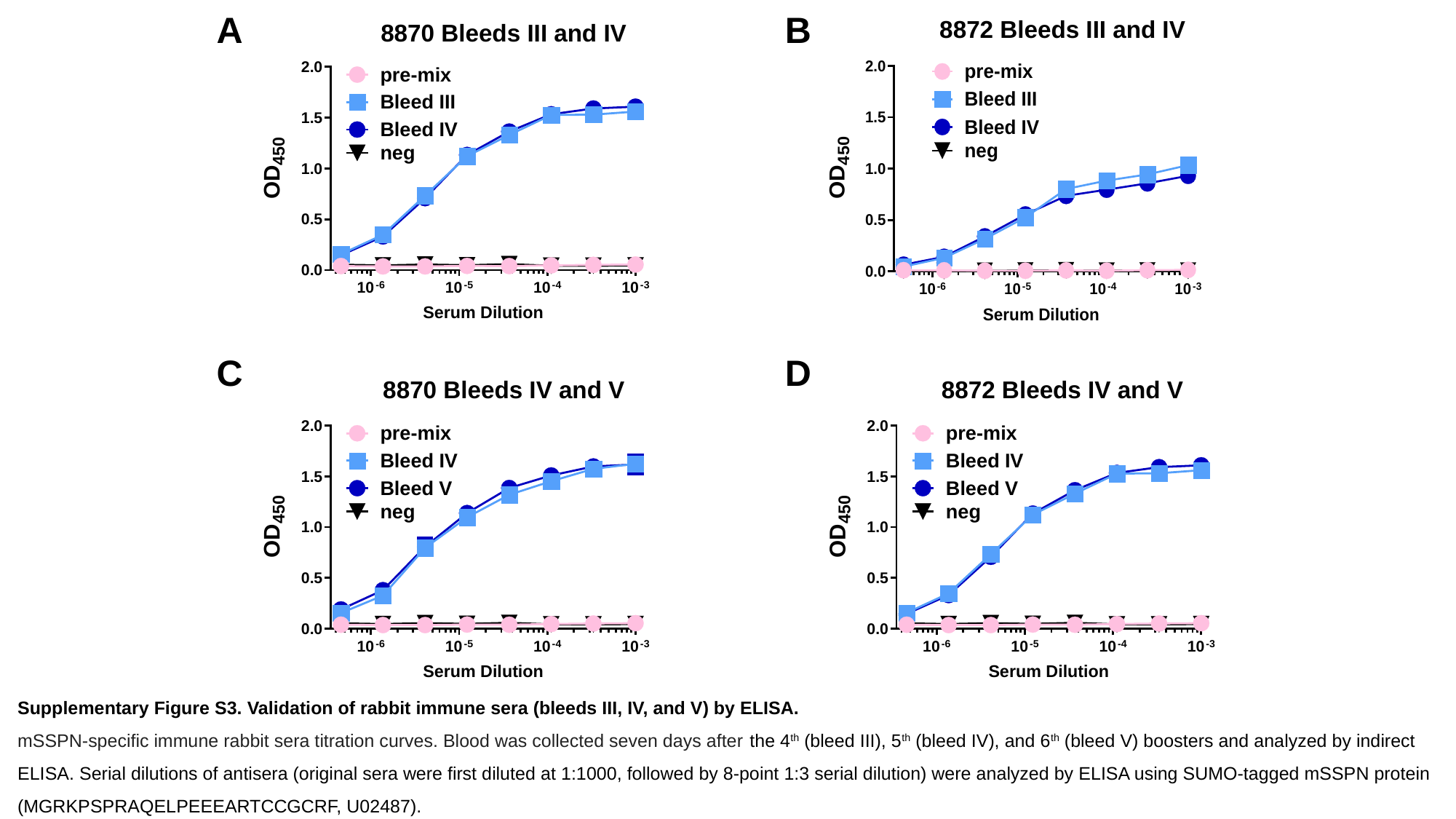

A
B
 8872 Bleeds III and IV
 8870 Bleeds III and IV
C
D
 8870 Bleeds IV and V
 8872 Bleeds IV and V
Supplementary Figure S3. Validation of rabbit immune sera (bleeds III, IV, and V) by ELISA.
mSSPN-specific immune rabbit sera titration curves. Blood was collected seven days after the 4th (bleed III), 5th (bleed IV), and 6th (bleed V) boosters and analyzed by indirect ELISA. Serial dilutions of antisera (original sera were first diluted at 1:1000, followed by 8-point 1:3 serial dilution) were analyzed by ELISA using SUMO-tagged mSSPN protein (MGRKPSPRAQELPEEEARTCCGCRF, U02487).

## Slide 6
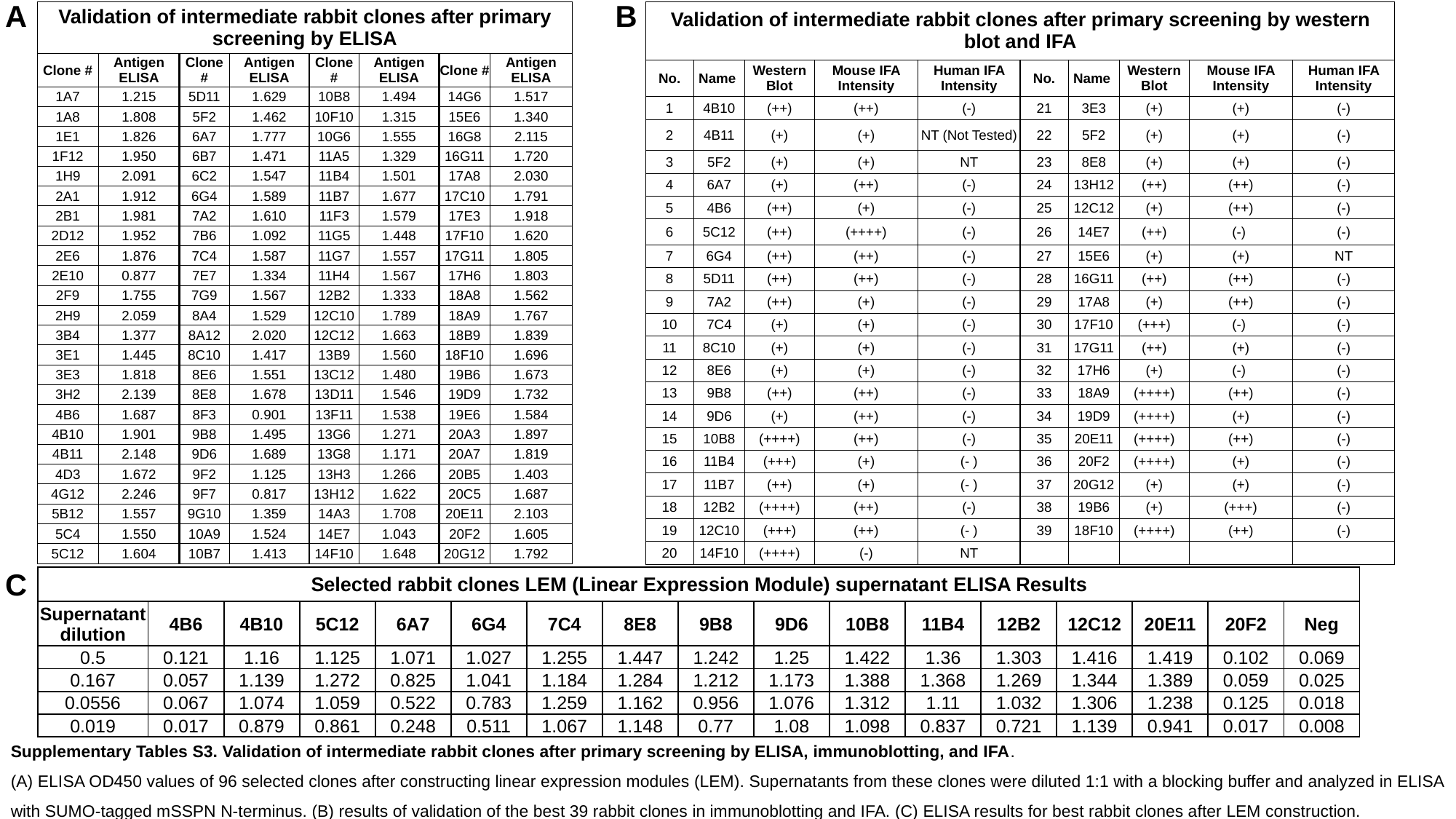

A
B
| Validation of intermediate rabbit clones after primary screening by ELISA | | | | | | | |
| --- | --- | --- | --- | --- | --- | --- | --- |
| Clone # | Antigen ELISA | Clone # | Antigen ELISA | Clone # | Antigen ELISA | Clone # | Antigen ELISA |
| 1A7 | 1.215 | 5D11 | 1.629 | 10B8 | 1.494 | 14G6 | 1.517 |
| 1A8 | 1.808 | 5F2 | 1.462 | 10F10 | 1.315 | 15E6 | 1.340 |
| 1E1 | 1.826 | 6A7 | 1.777 | 10G6 | 1.555 | 16G8 | 2.115 |
| 1F12 | 1.950 | 6B7 | 1.471 | 11A5 | 1.329 | 16G11 | 1.720 |
| 1H9 | 2.091 | 6C2 | 1.547 | 11B4 | 1.501 | 17A8 | 2.030 |
| 2A1 | 1.912 | 6G4 | 1.589 | 11B7 | 1.677 | 17C10 | 1.791 |
| 2B1 | 1.981 | 7A2 | 1.610 | 11F3 | 1.579 | 17E3 | 1.918 |
| 2D12 | 1.952 | 7B6 | 1.092 | 11G5 | 1.448 | 17F10 | 1.620 |
| 2E6 | 1.876 | 7C4 | 1.587 | 11G7 | 1.557 | 17G11 | 1.805 |
| 2E10 | 0.877 | 7E7 | 1.334 | 11H4 | 1.567 | 17H6 | 1.803 |
| 2F9 | 1.755 | 7G9 | 1.567 | 12B2 | 1.333 | 18A8 | 1.562 |
| 2H9 | 2.059 | 8A4 | 1.529 | 12C10 | 1.789 | 18A9 | 1.767 |
| 3B4 | 1.377 | 8A12 | 2.020 | 12C12 | 1.663 | 18B9 | 1.839 |
| 3E1 | 1.445 | 8C10 | 1.417 | 13B9 | 1.560 | 18F10 | 1.696 |
| 3E3 | 1.818 | 8E6 | 1.551 | 13C12 | 1.480 | 19B6 | 1.673 |
| 3H2 | 2.139 | 8E8 | 1.678 | 13D11 | 1.546 | 19D9 | 1.732 |
| 4B6 | 1.687 | 8F3 | 0.901 | 13F11 | 1.538 | 19E6 | 1.584 |
| 4B10 | 1.901 | 9B8 | 1.495 | 13G6 | 1.271 | 20A3 | 1.897 |
| 4B11 | 2.148 | 9D6 | 1.689 | 13G8 | 1.171 | 20A7 | 1.819 |
| 4D3 | 1.672 | 9F2 | 1.125 | 13H3 | 1.266 | 20B5 | 1.403 |
| 4G12 | 2.246 | 9F7 | 0.817 | 13H12 | 1.622 | 20C5 | 1.687 |
| 5B12 | 1.557 | 9G10 | 1.359 | 14A3 | 1.708 | 20E11 | 2.103 |
| 5C4 | 1.550 | 10A9 | 1.524 | 14E7 | 1.043 | 20F2 | 1.605 |
| 5C12 | 1.604 | 10B7 | 1.413 | 14F10 | 1.648 | 20G12 | 1.792 |
| Validation of intermediate rabbit clones after primary screening by western blot and IFA | | | | | | | | | |
| --- | --- | --- | --- | --- | --- | --- | --- | --- | --- |
| No. | Name | Western Blot | Mouse IFA Intensity | Human IFA Intensity | No. | Name | Western Blot | Mouse IFA Intensity | Human IFA Intensity |
| 1 | 4B10 | (++) | (++) | (-) | 21 | 3E3 | (+) | (+) | (-) |
| 2 | 4B11 | (+) | (+) | NT (Not Tested) | 22 | 5F2 | (+) | (+) | (-) |
| 3 | 5F2 | (+) | (+) | NT | 23 | 8E8 | (+) | (+) | (-) |
| 4 | 6A7 | (+) | (++) | (-) | 24 | 13H12 | (++) | (++) | (-) |
| 5 | 4B6 | (++) | (+) | (-) | 25 | 12C12 | (+) | (++) | (-) |
| 6 | 5C12 | (++) | (++++) | (-) | 26 | 14E7 | (++) | (-) | (-) |
| 7 | 6G4 | (++) | (++) | (-) | 27 | 15E6 | (+) | (+) | NT |
| 8 | 5D11 | (++) | (++) | (-) | 28 | 16G11 | (++) | (++) | (-) |
| 9 | 7A2 | (++) | (+) | (-) | 29 | 17A8 | (+) | (++) | (-) |
| 10 | 7C4 | (+) | (+) | (-) | 30 | 17F10 | (+++) | (-) | (-) |
| 11 | 8C10 | (+) | (+) | (-) | 31 | 17G11 | (++) | (+) | (-) |
| 12 | 8E6 | (+) | (+) | (-) | 32 | 17H6 | (+) | (-) | (-) |
| 13 | 9B8 | (++) | (++) | (-) | 33 | 18A9 | (++++) | (++) | (-) |
| 14 | 9D6 | (+) | (++) | (-) | 34 | 19D9 | (++++) | (+) | (-) |
| 15 | 10B8 | (++++) | (++) | (-) | 35 | 20E11 | (++++) | (++) | (-) |
| 16 | 11B4 | (+++) | (+) | (- ) | 36 | 20F2 | (++++) | (+) | (-) |
| 17 | 11B7 | (++) | (+) | (- ) | 37 | 20G12 | (+) | (+) | (-) |
| 18 | 12B2 | (++++) | (++) | (-) | 38 | 19B6 | (+) | (+++) | (-) |
| 19 | 12C10 | (+++) | (++) | (- ) | 39 | 18F10 | (++++) | (++) | (-) |
| 20 | 14F10 | (++++) | (-) | NT | | | | | |
C
| Selected rabbit clones LEM (Linear Expression Module) supernatant ELISA Results | | | | | | | | | | | | | | | | |
| --- | --- | --- | --- | --- | --- | --- | --- | --- | --- | --- | --- | --- | --- | --- | --- | --- |
| Supernatant dilution | 4B6 | 4B10 | 5C12 | 6A7 | 6G4 | 7C4 | 8E8 | 9B8 | 9D6 | 10B8 | 11B4 | 12B2 | 12C12 | 20E11 | 20F2 | Neg |
| 0.5 | 0.121 | 1.16 | 1.125 | 1.071 | 1.027 | 1.255 | 1.447 | 1.242 | 1.25 | 1.422 | 1.36 | 1.303 | 1.416 | 1.419 | 0.102 | 0.069 |
| 0.167 | 0.057 | 1.139 | 1.272 | 0.825 | 1.041 | 1.184 | 1.284 | 1.212 | 1.173 | 1.388 | 1.368 | 1.269 | 1.344 | 1.389 | 0.059 | 0.025 |
| 0.0556 | 0.067 | 1.074 | 1.059 | 0.522 | 0.783 | 1.259 | 1.162 | 0.956 | 1.076 | 1.312 | 1.11 | 1.032 | 1.306 | 1.238 | 0.125 | 0.018 |
| 0.019 | 0.017 | 0.879 | 0.861 | 0.248 | 0.511 | 1.067 | 1.148 | 0.77 | 1.08 | 1.098 | 0.837 | 0.721 | 1.139 | 0.941 | 0.017 | 0.008 |
Supplementary Tables S3. Validation of intermediate rabbit clones after primary screening by ELISA, immunoblotting, and IFA.
(A) ELISA OD450 values of 96 selected clones after constructing linear expression modules (LEM). Supernatants from these clones were diluted 1:1 with a blocking buffer and analyzed in ELISA with SUMO-tagged mSSPN N-terminus. (B) results of validation of the best 39 rabbit clones in immunoblotting and IFA. (C) ELISA results for best rabbit clones after LEM construction.

## Slide 7
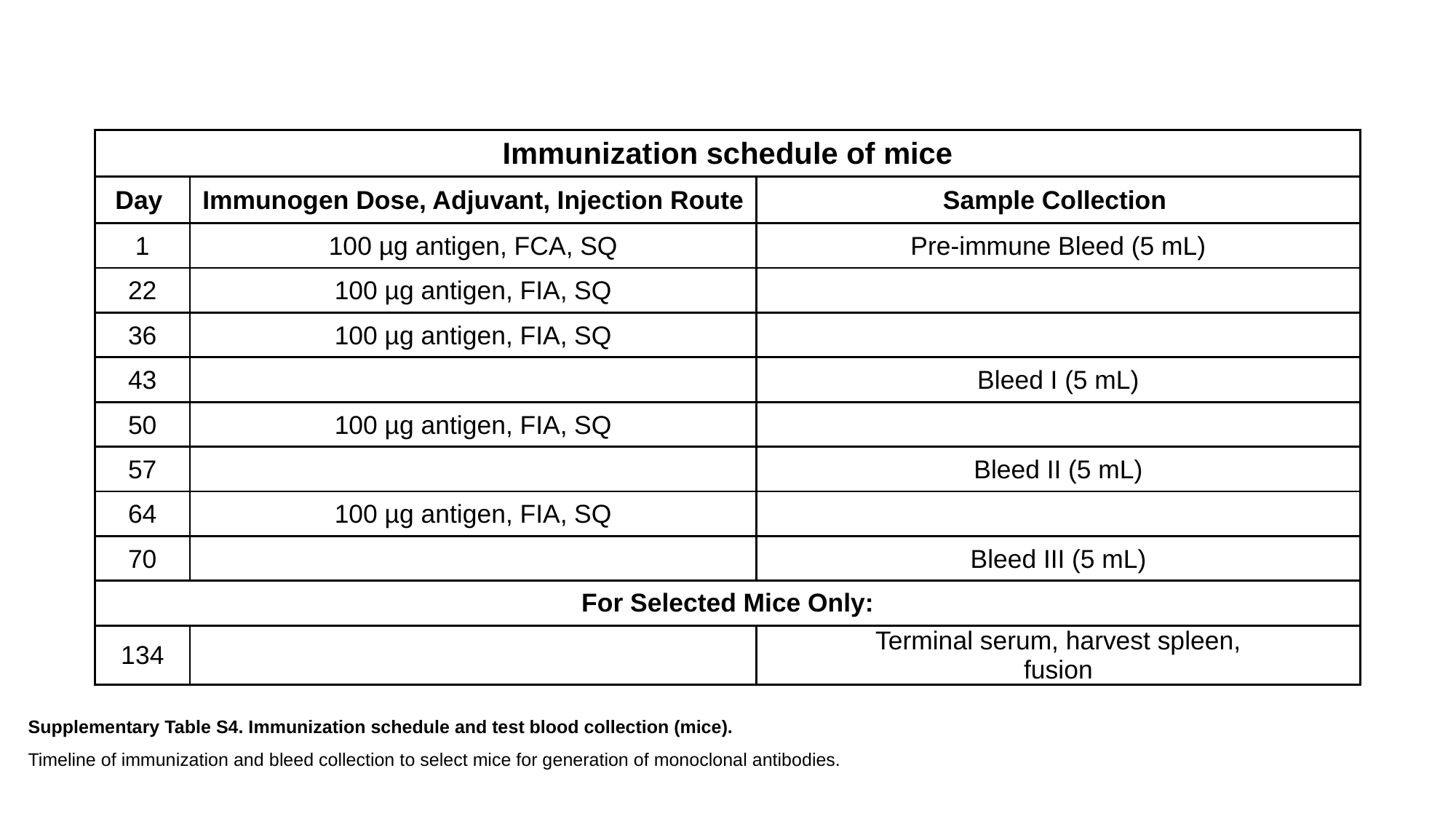

| Immunization schedule of mice | | |
| --- | --- | --- |
| Day | Immunogen Dose, Adjuvant, Injection Route | Sample Collection |
| 1 | 100 µg antigen, FCA, SQ | Pre-immune Bleed (5 mL) |
| 22 | 100 µg antigen, FIA, SQ | |
| 36 | 100 µg antigen, FIA, SQ | |
| 43 | | Bleed I (5 mL) |
| 50 | 100 µg antigen, FIA, SQ | |
| 57 | | Bleed II (5 mL) |
| 64 | 100 µg antigen, FIA, SQ | |
| 70 | | Bleed III (5 mL) |
| For Selected Mice Only: | | |
| 134 | | Terminal serum, harvest spleen, fusion |
Supplementary Table S4. Immunization schedule and test blood collection (mice).
Timeline of immunization and bleed collection to select mice for generation of monoclonal antibodies.

## Slide 8
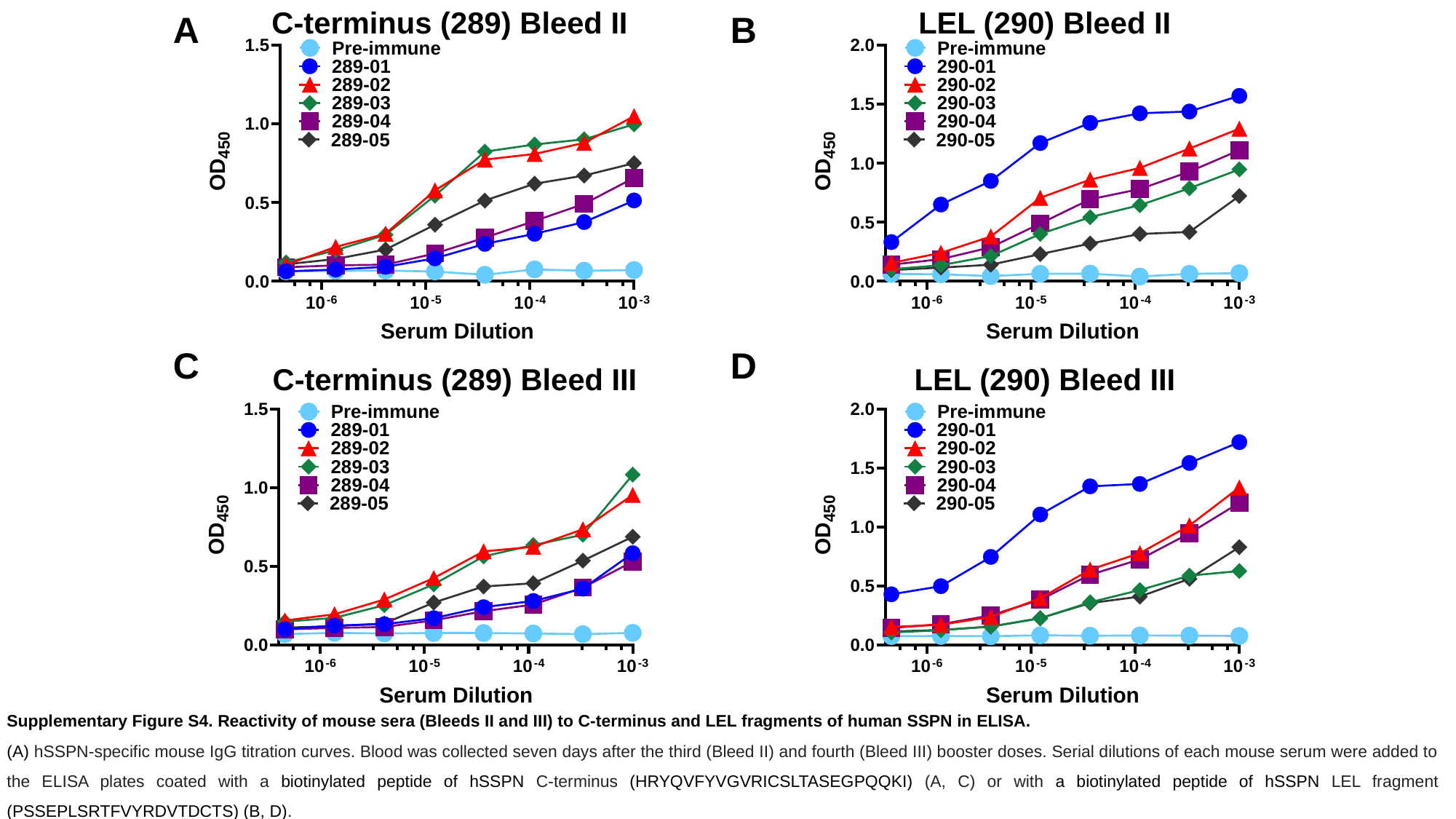

C-terminus (289) Bleed II
LEL (290) Bleed II
A
B
C
D
C-terminus (289) Bleed III
LEL (290) Bleed III
Supplementary Figure S4. Reactivity of mouse sera (Bleeds II and III) to C-terminus and LEL fragments of human SSPN in ELISA.
(A) hSSPN-specific mouse IgG titration curves. Blood was collected seven days after the third (Bleed II) and fourth (Bleed III) booster doses. Serial dilutions of each mouse serum were added to the ELISA plates coated with a biotinylated peptide of hSSPN C-terminus (HRYQVFYVGVRICSLTASEGPQQKI) (A, C) or with a biotinylated peptide of hSSPN LEL fragment (PSSEPLSRTFVYRDVTDCTS) (B, D).

## Slide 9
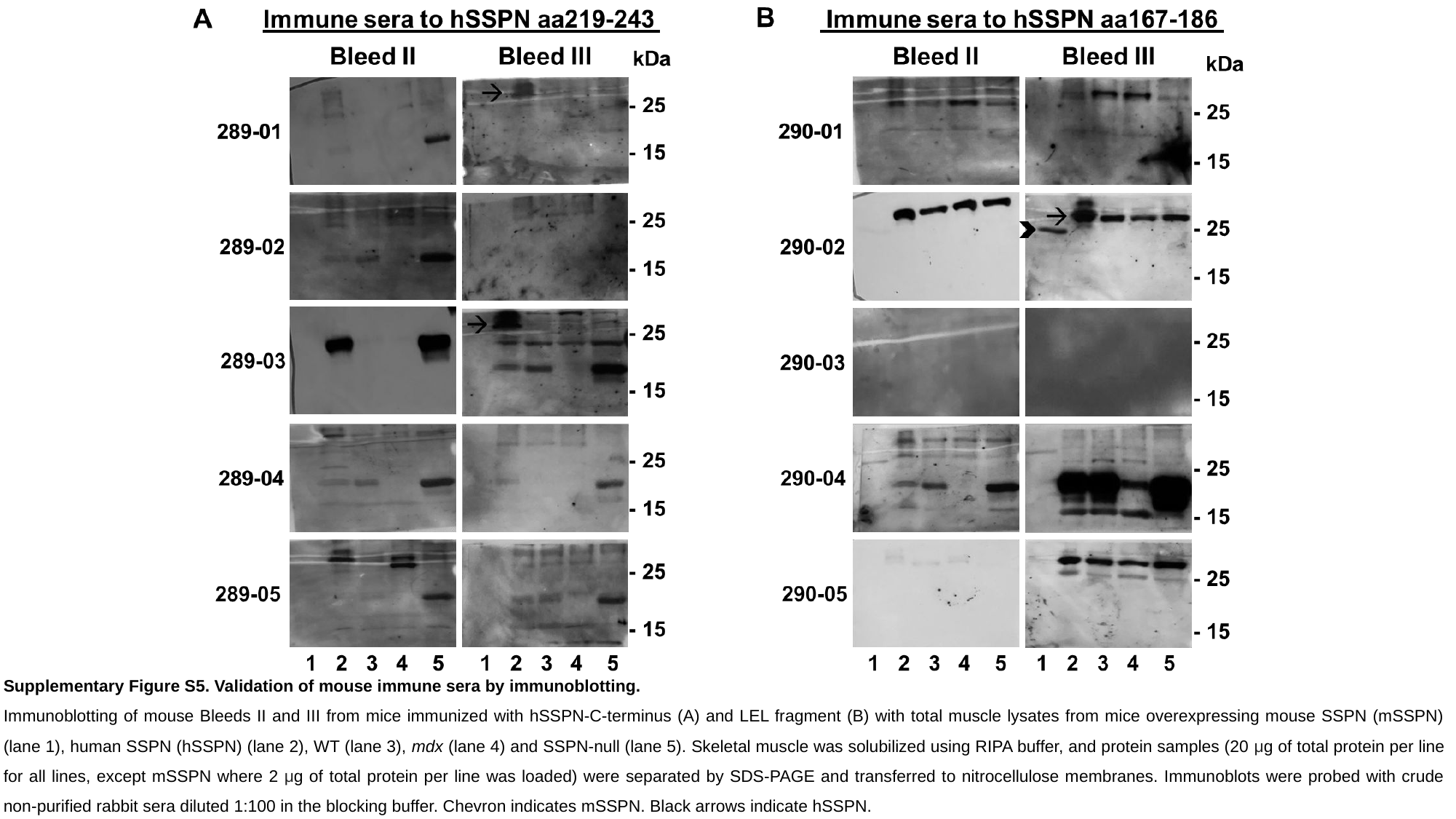

Supplementary Figure S5. Validation of mouse immune sera by immunoblotting.
Immunoblotting of mouse Bleeds II and III from mice immunized with hSSPN-C-terminus (A) and LEL fragment (B) with total muscle lysates from mice overexpressing mouse SSPN (mSSPN) (lane 1), human SSPN (hSSPN) (lane 2), WT (lane 3), mdx (lane 4) and SSPN-null (lane 5). Skeletal muscle was solubilized using RIPA buffer, and protein samples (20 μg of total protein per line for all lines, except mSSPN where 2 μg of total protein per line was loaded) were separated by SDS-PAGE and transferred to nitrocellulose membranes. Immunoblots were probed with crude non-purified rabbit sera diluted 1:100 in the blocking buffer. Chevron indicates mSSPN. Black arrows indicate hSSPN.

## Slide 10
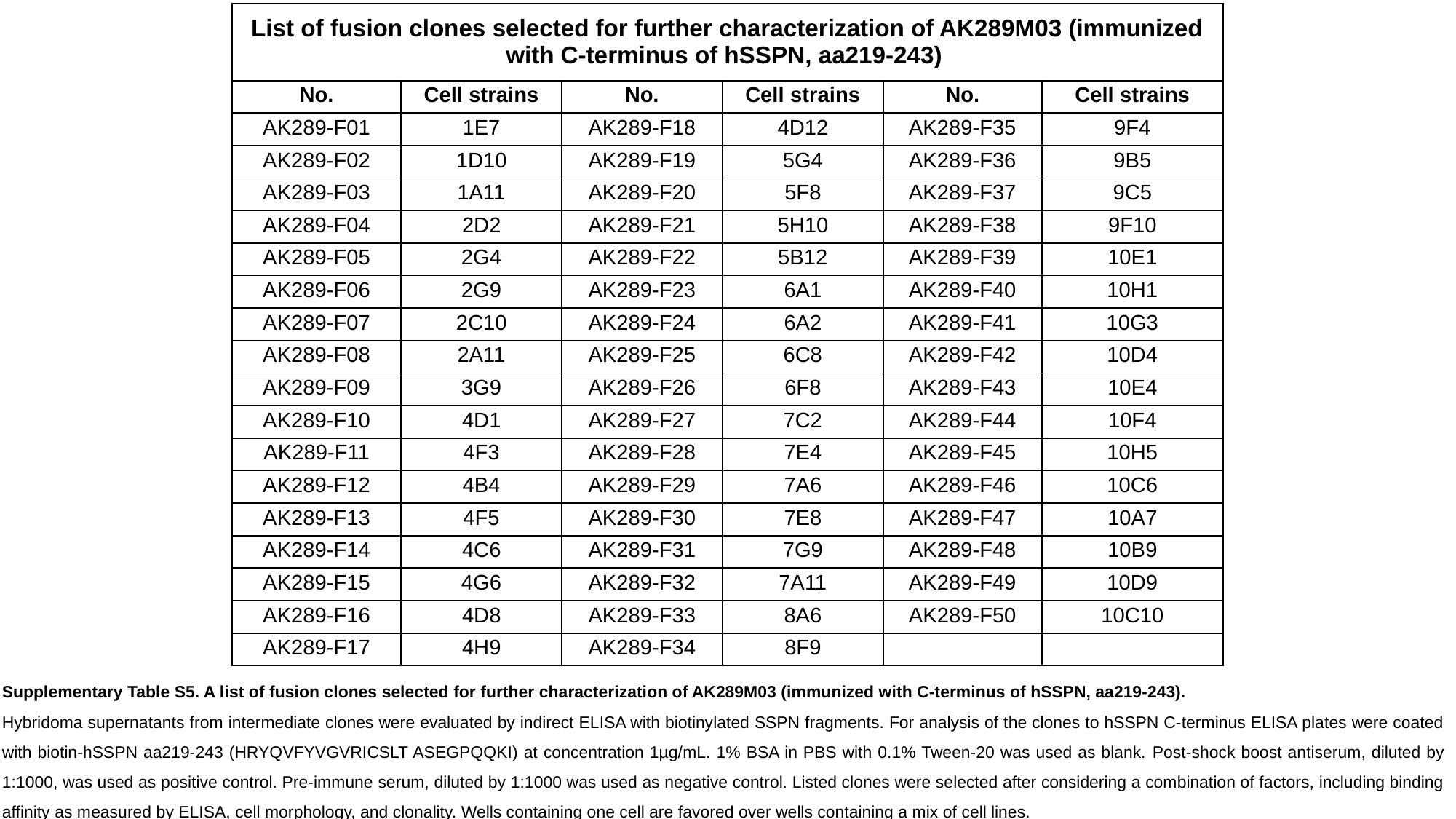

| List of fusion clones selected for further characterization of AK289M03 (immunized with C-terminus of hSSPN, aa219-243) | | | | | |
| --- | --- | --- | --- | --- | --- |
| No. | Cell strains | No. | Cell strains | No. | Cell strains |
| AK289-F01 | 1E7 | AK289-F18 | 4D12 | AK289-F35 | 9F4 |
| AK289-F02 | 1D10 | AK289-F19 | 5G4 | AK289-F36 | 9B5 |
| AK289-F03 | 1A11 | AK289-F20 | 5F8 | AK289-F37 | 9C5 |
| AK289-F04 | 2D2 | AK289-F21 | 5H10 | AK289-F38 | 9F10 |
| AK289-F05 | 2G4 | AK289-F22 | 5B12 | AK289-F39 | 10E1 |
| AK289-F06 | 2G9 | AK289-F23 | 6A1 | AK289-F40 | 10H1 |
| AK289-F07 | 2C10 | AK289-F24 | 6A2 | AK289-F41 | 10G3 |
| AK289-F08 | 2A11 | AK289-F25 | 6C8 | AK289-F42 | 10D4 |
| AK289-F09 | 3G9 | AK289-F26 | 6F8 | AK289-F43 | 10E4 |
| AK289-F10 | 4D1 | AK289-F27 | 7C2 | AK289-F44 | 10F4 |
| AK289-F11 | 4F3 | AK289-F28 | 7E4 | AK289-F45 | 10H5 |
| AK289-F12 | 4B4 | AK289-F29 | 7A6 | AK289-F46 | 10C6 |
| AK289-F13 | 4F5 | AK289-F30 | 7E8 | AK289-F47 | 10A7 |
| AK289-F14 | 4C6 | AK289-F31 | 7G9 | AK289-F48 | 10B9 |
| AK289-F15 | 4G6 | AK289-F32 | 7A11 | AK289-F49 | 10D9 |
| AK289-F16 | 4D8 | AK289-F33 | 8A6 | AK289-F50 | 10C10 |
| AK289-F17 | 4H9 | AK289-F34 | 8F9 | | |
Supplementary Table S5. A list of fusion clones selected for further characterization of AK289M03 (immunized with C-terminus of hSSPN, aa219-243).
Hybridoma supernatants from intermediate clones were evaluated by indirect ELISA with biotinylated SSPN fragments. For analysis of the clones to hSSPN C-terminus ELISA plates were coated with biotin-hSSPN aa219-243 (HRYQVFYVGVRICSLT ASEGPQQKI) at concentration 1µg/mL. 1% BSA in PBS with 0.1% Tween-20 was used as blank. Post-shock boost antiserum, diluted by 1:1000, was used as positive control. Pre-immune serum, diluted by 1:1000 was used as negative control. Listed clones were selected after considering a combination of factors, including binding affinity as measured by ELISA, cell morphology, and clonality. Wells containing one cell are favored over wells containing a mix of cell lines.

## Slide 11
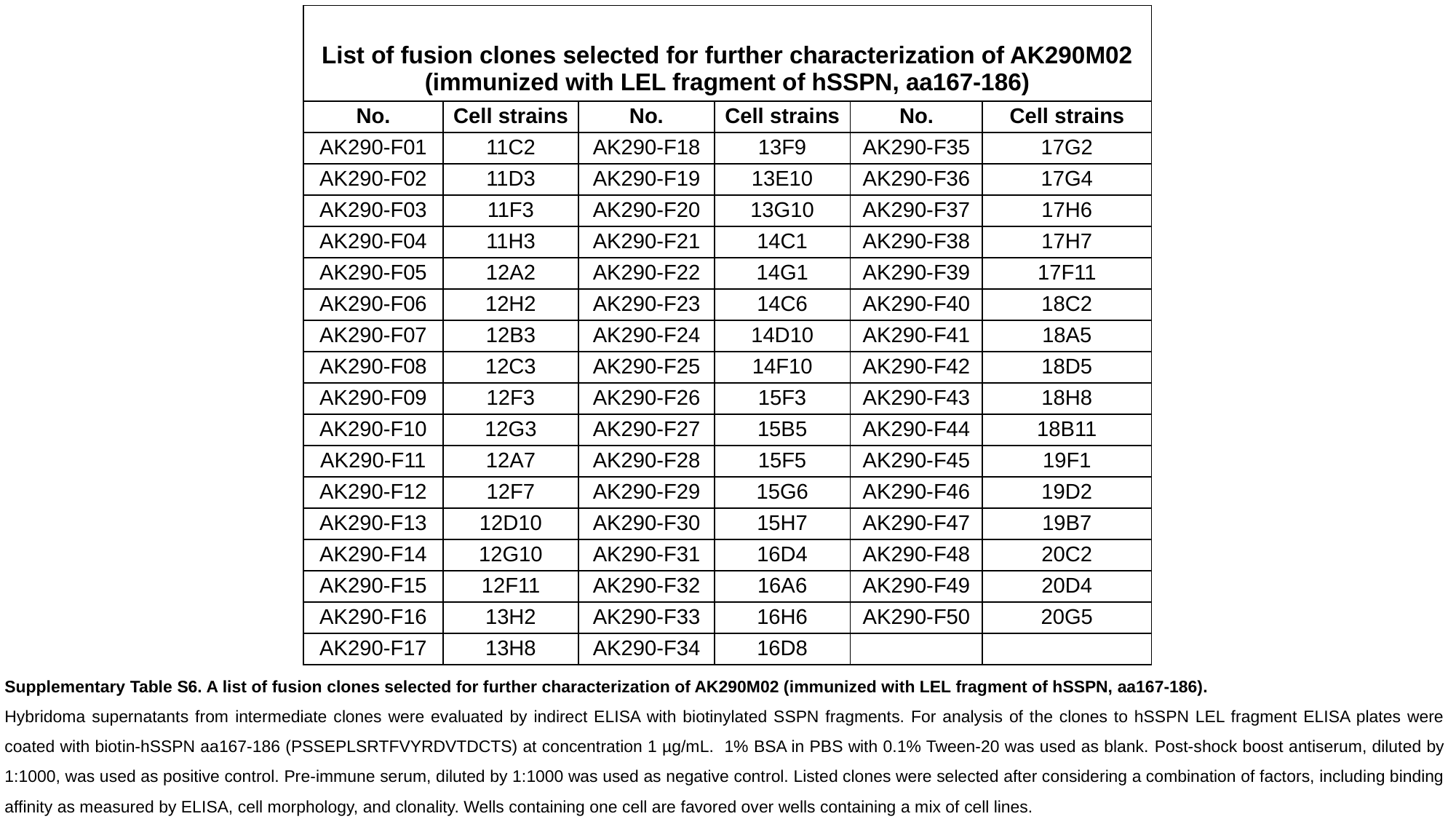

| List of fusion clones selected for further characterization of AK290M02 (immunized with LEL fragment of hSSPN, aa167-186) | | | | | |
| --- | --- | --- | --- | --- | --- |
| No. | Cell strains | No. | Cell strains | No. | Cell strains |
| AK290-F01 | 11C2 | AK290-F18 | 13F9 | AK290-F35 | 17G2 |
| AK290-F02 | 11D3 | AK290-F19 | 13E10 | AK290-F36 | 17G4 |
| AK290-F03 | 11F3 | AK290-F20 | 13G10 | AK290-F37 | 17H6 |
| AK290-F04 | 11H3 | AK290-F21 | 14C1 | AK290-F38 | 17H7 |
| AK290-F05 | 12A2 | AK290-F22 | 14G1 | AK290-F39 | 17F11 |
| AK290-F06 | 12H2 | AK290-F23 | 14C6 | AK290-F40 | 18C2 |
| AK290-F07 | 12B3 | AK290-F24 | 14D10 | AK290-F41 | 18A5 |
| AK290-F08 | 12C3 | AK290-F25 | 14F10 | AK290-F42 | 18D5 |
| AK290-F09 | 12F3 | AK290-F26 | 15F3 | AK290-F43 | 18H8 |
| AK290-F10 | 12G3 | AK290-F27 | 15B5 | AK290-F44 | 18B11 |
| AK290-F11 | 12A7 | AK290-F28 | 15F5 | AK290-F45 | 19F1 |
| AK290-F12 | 12F7 | AK290-F29 | 15G6 | AK290-F46 | 19D2 |
| AK290-F13 | 12D10 | AK290-F30 | 15H7 | AK290-F47 | 19B7 |
| AK290-F14 | 12G10 | AK290-F31 | 16D4 | AK290-F48 | 20C2 |
| AK290-F15 | 12F11 | AK290-F32 | 16A6 | AK290-F49 | 20D4 |
| AK290-F16 | 13H2 | AK290-F33 | 16H6 | AK290-F50 | 20G5 |
| AK290-F17 | 13H8 | AK290-F34 | 16D8 | | |
Supplementary Table S6. A list of fusion clones selected for further characterization of AK290M02 (immunized with LEL fragment of hSSPN, aa167-186).
Hybridoma supernatants from intermediate clones were evaluated by indirect ELISA with biotinylated SSPN fragments. For analysis of the clones to hSSPN LEL fragment ELISA plates were coated with biotin-hSSPN aa167-186 (PSSEPLSRTFVYRDVTDCTS) at concentration 1 µg/mL. 1% BSA in PBS with 0.1% Tween-20 was used as blank. Post-shock boost antiserum, diluted by 1:1000, was used as positive control. Pre-immune serum, diluted by 1:1000 was used as negative control. Listed clones were selected after considering a combination of factors, including binding affinity as measured by ELISA, cell morphology, and clonality. Wells containing one cell are favored over wells containing a mix of cell lines.

## Slide 12
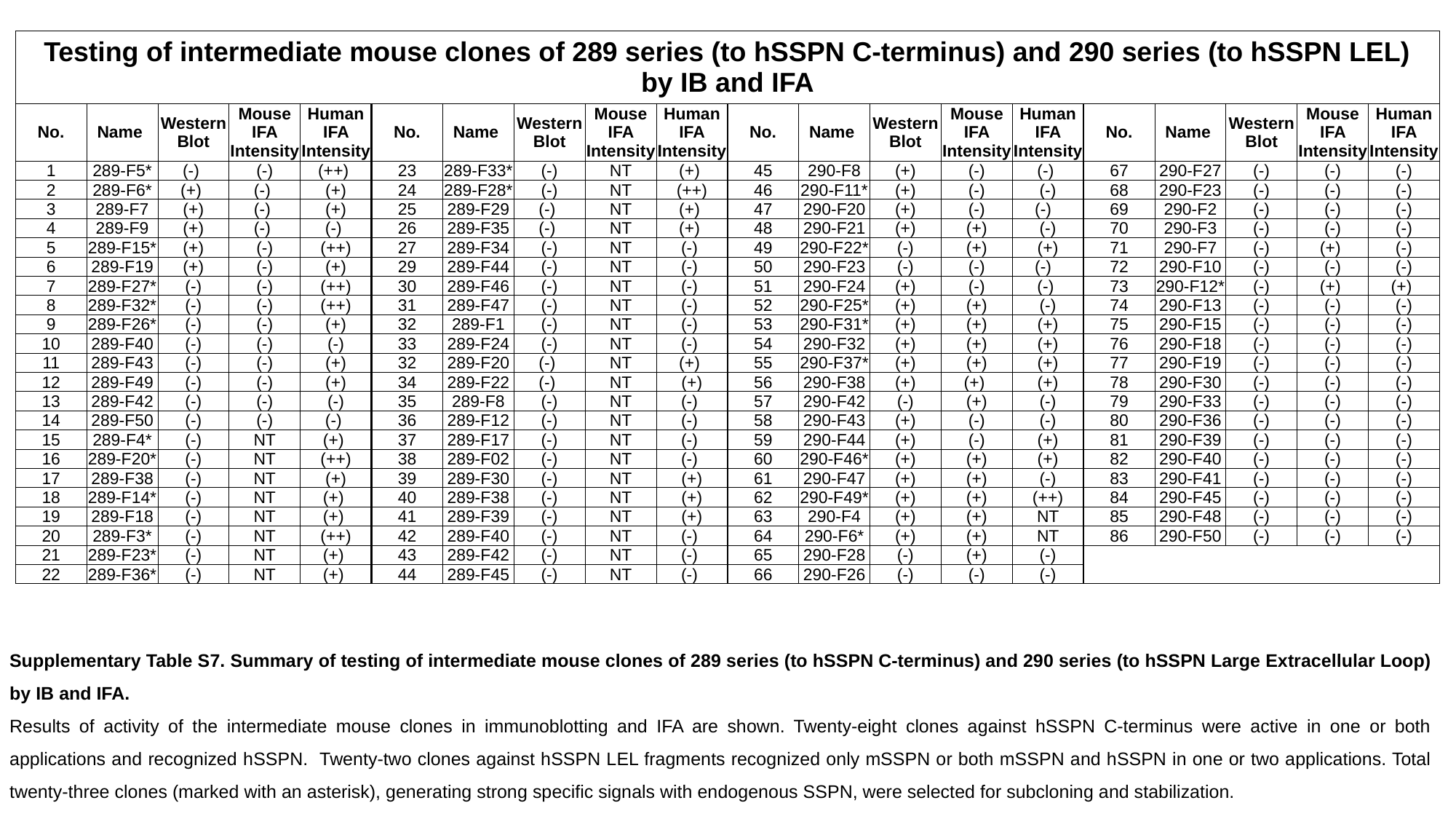

| Testing of intermediate mouse clones of 289 series (to hSSPN C-terminus) and 290 series (to hSSPN LEL) by IB and IFA | | | | | | | | | | | | | | | | | | | |
| --- | --- | --- | --- | --- | --- | --- | --- | --- | --- | --- | --- | --- | --- | --- | --- | --- | --- | --- | --- |
| No. | Name | Western Blot | Mouse IFA Intensity | Human IFA Intensity | No. | Name | Western Blot | Mouse IFA Intensity | Human IFA Intensity | No. | Name | Western Blot | Mouse IFA Intensity | Human IFA Intensity | No. | Name | Western Blot | Mouse IFA Intensity | Human IFA Intensity |
| 1 | 289-F5\* | (-) | (-) | (++) | 23 | 289-F33\* | (-) | NT | (+) | 45 | 290-F8 | (+) | (-) | (-) | 67 | 290-F27 | (-) | (-) | (-) |
| 2 | 289-F6\* | (+) | (-) | (+) | 24 | 289-F28\* | (-) | NT | (++) | 46 | 290-F11\* | (+) | (-) | (-) | 68 | 290-F23 | (-) | (-) | (-) |
| 3 | 289-F7 | (+) | (-) | (+) | 25 | 289-F29 | (-) | NT | (+) | 47 | 290-F20 | (+) | (-) | (-) | 69 | 290-F2 | (-) | (-) | (-) |
| 4 | 289-F9 | (+) | (-) | (-) | 26 | 289-F35 | (-) | NT | (+) | 48 | 290-F21 | (+) | (+) | (-) | 70 | 290-F3 | (-) | (-) | (-) |
| 5 | 289-F15\* | (+) | (-) | (++) | 27 | 289-F34 | (-) | NT | (-) | 49 | 290-F22\* | (-) | (+) | (+) | 71 | 290-F7 | (-) | (+) | (-) |
| 6 | 289-F19 | (+) | (-) | (+) | 29 | 289-F44 | (-) | NT | (-) | 50 | 290-F23 | (-) | (-) | (-) | 72 | 290-F10 | (-) | (-) | (-) |
| 7 | 289-F27\* | (-) | (-) | (++) | 30 | 289-F46 | (-) | NT | (-) | 51 | 290-F24 | (+) | (-) | (-) | 73 | 290-F12\* | (-) | (+) | (+) |
| 8 | 289-F32\* | (-) | (-) | (++) | 31 | 289-F47 | (-) | NT | (-) | 52 | 290-F25\* | (+) | (+) | (-) | 74 | 290-F13 | (-) | (-) | (-) |
| 9 | 289-F26\* | (-) | (-) | (+) | 32 | 289-F1 | (-) | NT | (-) | 53 | 290-F31\* | (+) | (+) | (+) | 75 | 290-F15 | (-) | (-) | (-) |
| 10 | 289-F40 | (-) | (-) | (-) | 33 | 289-F24 | (-) | NT | (-) | 54 | 290-F32 | (+) | (+) | (+) | 76 | 290-F18 | (-) | (-) | (-) |
| 11 | 289-F43 | (-) | (-) | (+) | 32 | 289-F20 | (-) | NT | (+) | 55 | 290-F37\* | (+) | (+) | (+) | 77 | 290-F19 | (-) | (-) | (-) |
| 12 | 289-F49 | (-) | (-) | (+) | 34 | 289-F22 | (-) | NT | (+) | 56 | 290-F38 | (+) | (+) | (+) | 78 | 290-F30 | (-) | (-) | (-) |
| 13 | 289-F42 | (-) | (-) | (-) | 35 | 289-F8 | (-) | NT | (-) | 57 | 290-F42 | (-) | (+) | (-) | 79 | 290-F33 | (-) | (-) | (-) |
| 14 | 289-F50 | (-) | (-) | (-) | 36 | 289-F12 | (-) | NT | (-) | 58 | 290-F43 | (+) | (-) | (-) | 80 | 290-F36 | (-) | (-) | (-) |
| 15 | 289-F4\* | (-) | NT | (+) | 37 | 289-F17 | (-) | NT | (-) | 59 | 290-F44 | (+) | (-) | (+) | 81 | 290-F39 | (-) | (-) | (-) |
| 16 | 289-F20\* | (-) | NT | (++) | 38 | 289-F02 | (-) | NT | (-) | 60 | 290-F46\* | (+) | (+) | (+) | 82 | 290-F40 | (-) | (-) | (-) |
| 17 | 289-F38 | (-) | NT | (+) | 39 | 289-F30 | (-) | NT | (+) | 61 | 290-F47 | (+) | (+) | (-) | 83 | 290-F41 | (-) | (-) | (-) |
| 18 | 289-F14\* | (-) | NT | (+) | 40 | 289-F38 | (-) | NT | (+) | 62 | 290-F49\* | (+) | (+) | (++) | 84 | 290-F45 | (-) | (-) | (-) |
| 19 | 289-F18 | (-) | NT | (+) | 41 | 289-F39 | (-) | NT | (+) | 63 | 290-F4 | (+) | (+) | NT | 85 | 290-F48 | (-) | (-) | (-) |
| 20 | 289-F3\* | (-) | NT | (++) | 42 | 289-F40 | (-) | NT | (-) | 64 | 290-F6\* | (+) | (+) | NT | 86 | 290-F50 | (-) | (-) | (-) |
| 21 | 289-F23\* | (-) | NT | (+) | 43 | 289-F42 | (-) | NT | (-) | 65 | 290-F28 | (-) | (+) | (-) | | | | | |
| 22 | 289-F36\* | (-) | NT | (+) | 44 | 289-F45 | (-) | NT | (-) | 66 | 290-F26 | (-) | (-) | (-) | | | | | |
Supplementary Table S7. Summary of testing of intermediate mouse clones of 289 series (to hSSPN C-terminus) and 290 series (to hSSPN Large Extracellular Loop) by IB and IFA.
Results of activity of the intermediate mouse clones in immunoblotting and IFA are shown. Twenty-eight clones against hSSPN C-terminus were active in one or both applications and recognized hSSPN. Twenty-two clones against hSSPN LEL fragments recognized only mSSPN or both mSSPN and hSSPN in one or two applications. Total twenty-three clones (marked with an asterisk), generating strong specific signals with endogenous SSPN, were selected for subcloning and stabilization.

## Slide 13
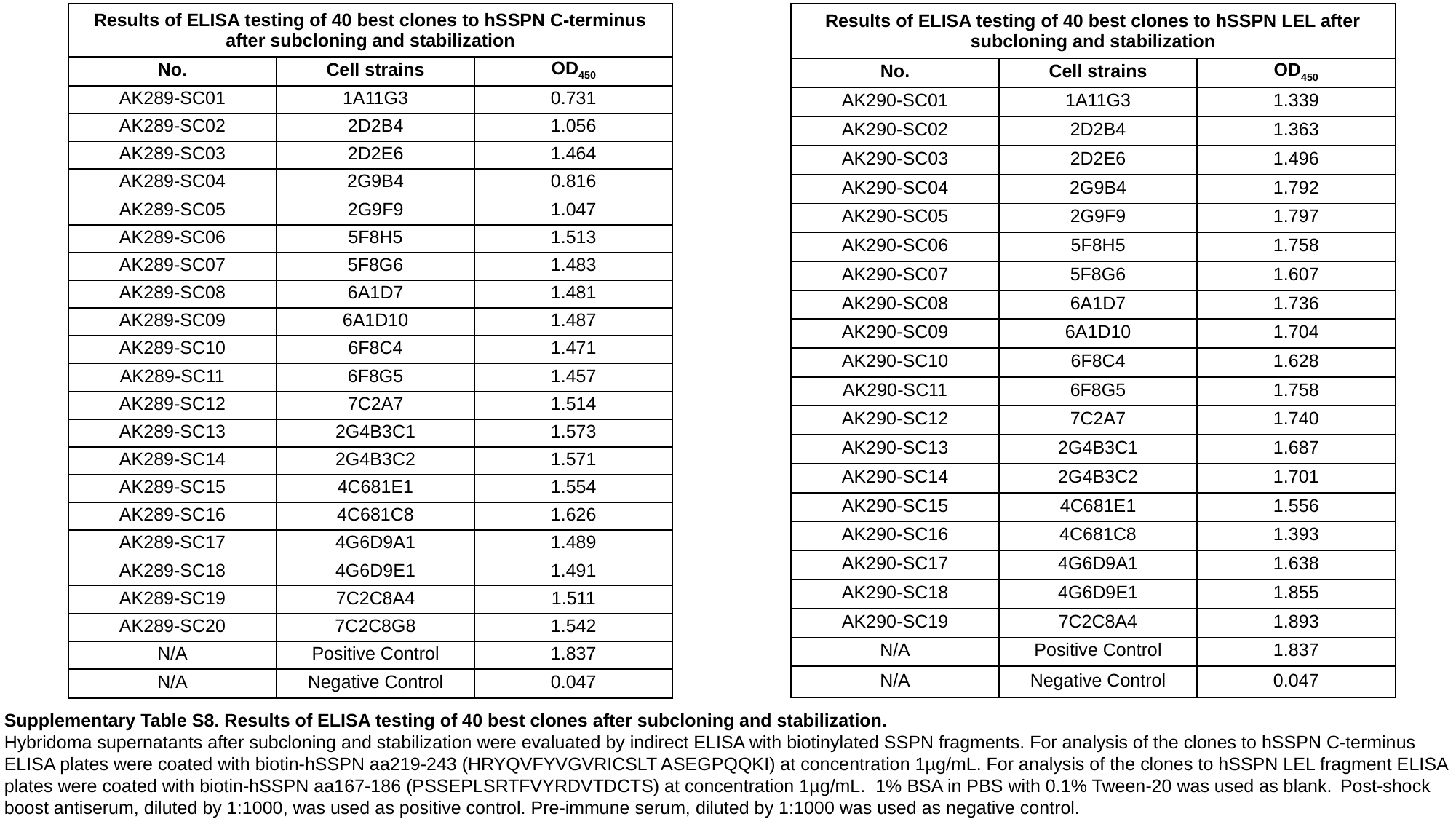

| Results of ELISA testing of 40 best clones to hSSPN C-terminus after subcloning and stabilization | | |
| --- | --- | --- |
| No. | Cell strains | OD450 |
| AK289-SC01 | 1A11G3 | 0.731 |
| AK289-SC02 | 2D2B4 | 1.056 |
| AK289-SC03 | 2D2E6 | 1.464 |
| AK289-SC04 | 2G9B4 | 0.816 |
| AK289-SC05 | 2G9F9 | 1.047 |
| AK289-SC06 | 5F8H5 | 1.513 |
| AK289-SC07 | 5F8G6 | 1.483 |
| AK289-SC08 | 6A1D7 | 1.481 |
| AK289-SC09 | 6A1D10 | 1.487 |
| AK289-SC10 | 6F8C4 | 1.471 |
| AK289-SC11 | 6F8G5 | 1.457 |
| AK289-SC12 | 7C2A7 | 1.514 |
| AK289-SC13 | 2G4B3C1 | 1.573 |
| AK289-SC14 | 2G4B3C2 | 1.571 |
| AK289-SC15 | 4C681E1 | 1.554 |
| AK289-SC16 | 4C681C8 | 1.626 |
| AK289-SC17 | 4G6D9A1 | 1.489 |
| AK289-SC18 | 4G6D9E1 | 1.491 |
| AK289-SC19 | 7C2C8A4 | 1.511 |
| AK289-SC20 | 7C2C8G8 | 1.542 |
| N/A | Positive Control | 1.837 |
| N/A | Negative Control | 0.047 |
| Results of ELISA testing of 40 best clones to hSSPN LEL after subcloning and stabilization | | |
| --- | --- | --- |
| No. | Cell strains | OD450 |
| AK290-SC01 | 1A11G3 | 1.339 |
| AK290-SC02 | 2D2B4 | 1.363 |
| AK290-SC03 | 2D2E6 | 1.496 |
| AK290-SC04 | 2G9B4 | 1.792 |
| AK290-SC05 | 2G9F9 | 1.797 |
| AK290-SC06 | 5F8H5 | 1.758 |
| AK290-SC07 | 5F8G6 | 1.607 |
| AK290-SC08 | 6A1D7 | 1.736 |
| AK290-SC09 | 6A1D10 | 1.704 |
| AK290-SC10 | 6F8C4 | 1.628 |
| AK290-SC11 | 6F8G5 | 1.758 |
| AK290-SC12 | 7C2A7 | 1.740 |
| AK290-SC13 | 2G4B3C1 | 1.687 |
| AK290-SC14 | 2G4B3C2 | 1.701 |
| AK290-SC15 | 4C681E1 | 1.556 |
| AK290-SC16 | 4C681C8 | 1.393 |
| AK290-SC17 | 4G6D9A1 | 1.638 |
| AK290-SC18 | 4G6D9E1 | 1.855 |
| AK290-SC19 | 7C2C8A4 | 1.893 |
| N/A | Positive Control | 1.837 |
| N/A | Negative Control | 0.047 |
Supplementary Table S8. Results of ELISA testing of 40 best clones after subcloning and stabilization.
Hybridoma supernatants after subcloning and stabilization were evaluated by indirect ELISA with biotinylated SSPN fragments. For analysis of the clones to hSSPN C-terminus ELISA plates were coated with biotin-hSSPN aa219-243 (HRYQVFYVGVRICSLT ASEGPQQKI) at concentration 1µg/mL. For analysis of the clones to hSSPN LEL fragment ELISA plates were coated with biotin-hSSPN aa167-186 (PSSEPLSRTFVYRDVTDCTS) at concentration 1µg/mL. 1% BSA in PBS with 0.1% Tween-20 was used as blank. Post-shock boost antiserum, diluted by 1:1000, was used as positive control. Pre-immune serum, diluted by 1:1000 was used as negative control.

## Slide 14
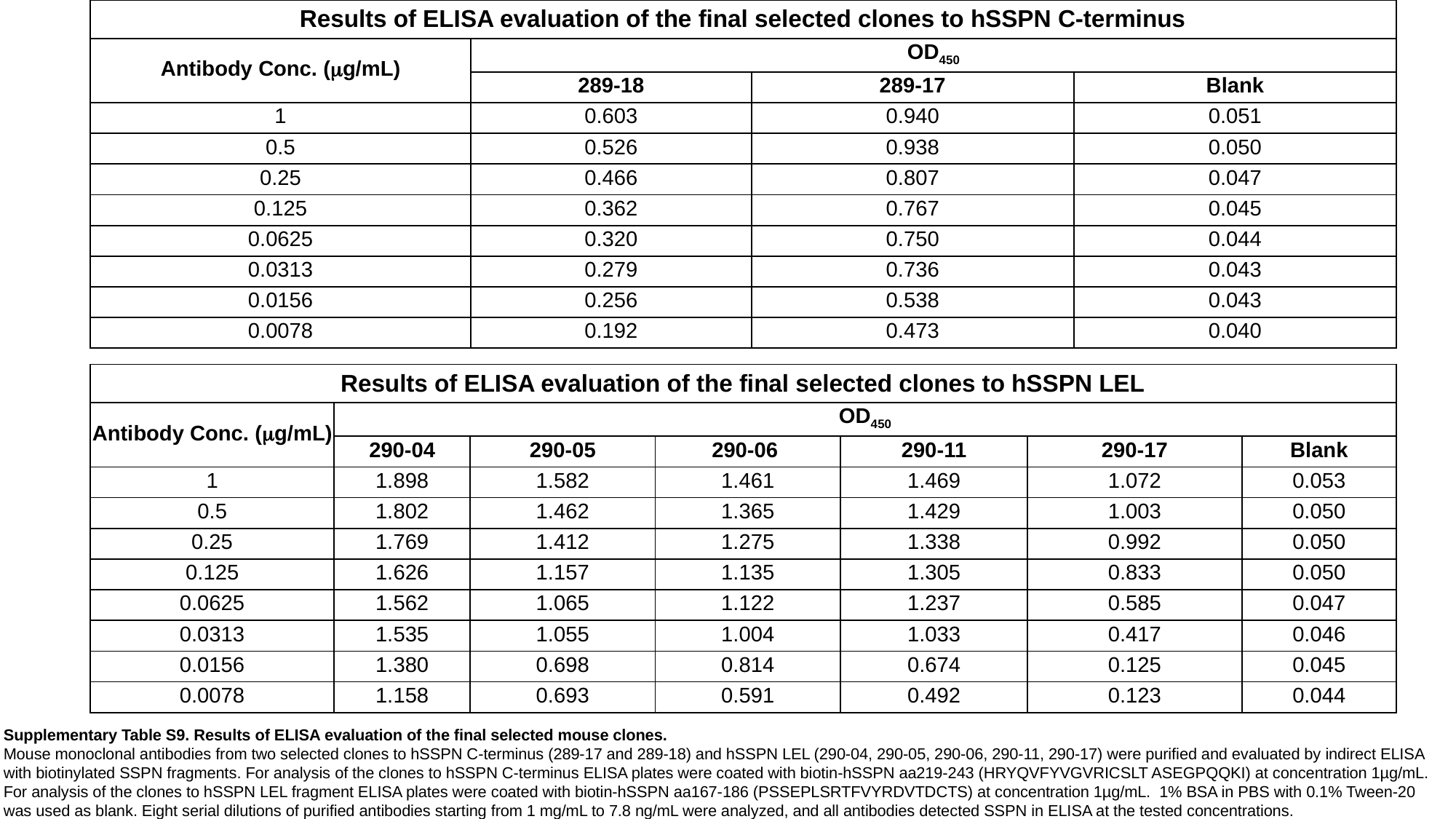

| Results of ELISA evaluation of the final selected clones to hSSPN C-terminus | | | |
| --- | --- | --- | --- |
| Antibody Conc. (mg/mL) | OD450 | OD (450nm) | OD (450nm) |
| Antibody Conc. (ug/ml) | 289-18 | 289-17 | Blank |
| 1 | 0.603 | 0.940 | 0.051 |
| 0.5 | 0.526 | 0.938 | 0.050 |
| 0.25 | 0.466 | 0.807 | 0.047 |
| 0.125 | 0.362 | 0.767 | 0.045 |
| 0.0625 | 0.320 | 0.750 | 0.044 |
| 0.0313 | 0.279 | 0.736 | 0.043 |
| 0.0156 | 0.256 | 0.538 | 0.043 |
| 0.0078 | 0.192 | 0.473 | 0.040 |
| Results of ELISA evaluation of the final selected clones to hSSPN LEL | | | | | | |
| --- | --- | --- | --- | --- | --- | --- |
| Antibody Conc. (mg/mL) | OD450 | | | | | |
| Antibody Conc. (ug/ml) | 290-04 | 290-05 | 290-06 | 290-11 | 290-17 | Blank |
| 1 | 1.898 | 1.582 | 1.461 | 1.469 | 1.072 | 0.053 |
| 0.5 | 1.802 | 1.462 | 1.365 | 1.429 | 1.003 | 0.050 |
| 0.25 | 1.769 | 1.412 | 1.275 | 1.338 | 0.992 | 0.050 |
| 0.125 | 1.626 | 1.157 | 1.135 | 1.305 | 0.833 | 0.050 |
| 0.0625 | 1.562 | 1.065 | 1.122 | 1.237 | 0.585 | 0.047 |
| 0.0313 | 1.535 | 1.055 | 1.004 | 1.033 | 0.417 | 0.046 |
| 0.0156 | 1.380 | 0.698 | 0.814 | 0.674 | 0.125 | 0.045 |
| 0.0078 | 1.158 | 0.693 | 0.591 | 0.492 | 0.123 | 0.044 |
Supplementary Table S9. Results of ELISA evaluation of the final selected mouse clones.
Mouse monoclonal antibodies from two selected clones to hSSPN C-terminus (289-17 and 289-18) and hSSPN LEL (290-04, 290-05, 290-06, 290-11, 290-17) were purified and evaluated by indirect ELISA with biotinylated SSPN fragments. For analysis of the clones to hSSPN C-terminus ELISA plates were coated with biotin-hSSPN aa219-243 (HRYQVFYVGVRICSLT ASEGPQQKI) at concentration 1µg/mL. For analysis of the clones to hSSPN LEL fragment ELISA plates were coated with biotin-hSSPN aa167-186 (PSSEPLSRTFVYRDVTDCTS) at concentration 1µg/mL. 1% BSA in PBS with 0.1% Tween-20 was used as blank. Eight serial dilutions of purified antibodies starting from 1 mg/mL to 7.8 ng/mL were analyzed, and all antibodies detected SSPN in ELISA at the tested concentrations.

## Slide 15
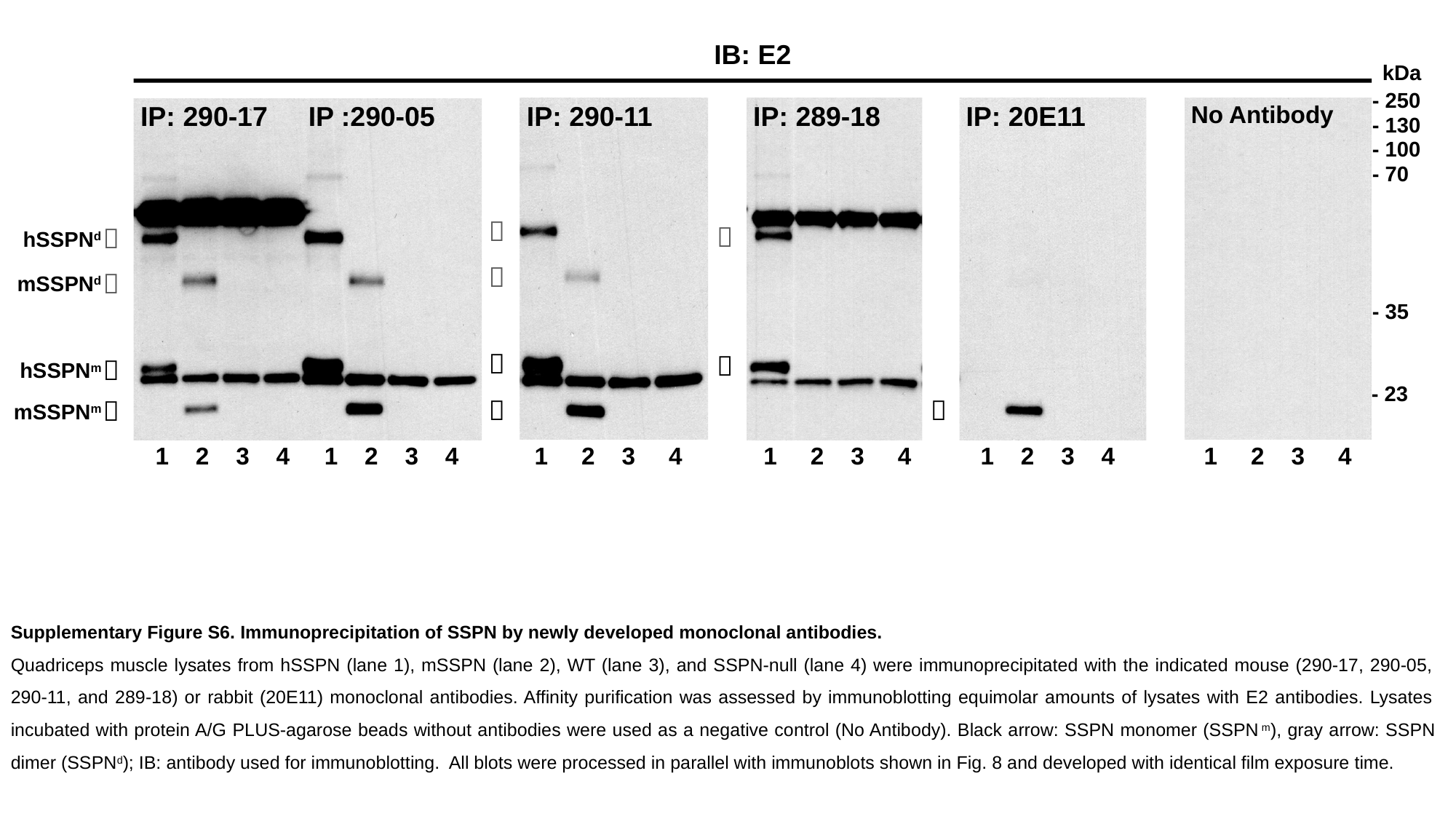

IB: E2
kDa
- 250
IP: 290-17
IP :290-05
IP: 290-11
IP: 289-18
IP: 20E11
No Antibody
- 130
- 100
- 70



hSSPNd


mSSPNd
- 35



hSSPNm
- 23



mSSPNm
 1 2 3 4
 1 2 3 4
1 2 3 4
1 2 3 4
 1 2 3 4
1 2 3 4
Supplementary Figure S6. Immunoprecipitation of SSPN by newly developed monoclonal antibodies.
Quadriceps muscle lysates from hSSPN (lane 1), mSSPN (lane 2), WT (lane 3), and SSPN-null (lane 4) were immunoprecipitated with the indicated mouse (290-17, 290-05, 290-11, and 289-18) or rabbit (20E11) monoclonal antibodies. Affinity purification was assessed by immunoblotting equimolar amounts of lysates with E2 antibodies. Lysates incubated with protein A/G PLUS-agarose beads without antibodies were used as a negative control (No Antibody). Black arrow: SSPN monomer (SSPNm), gray arrow: SSPN dimer (SSPNd); IB: antibody used for immunoblotting.  All blots were processed in parallel with immunoblots shown in Fig. 8 and developed with identical film exposure time.
